# Supplementary material for: Tracking the coherent generation of polaron pairs in conjugated polymers
Source: Nat Commun. 2016 Dec 8;7:13742. doi: 10.1038/ncomms13742 (PMC5155154; doi:10.1038/ncomms13742)
Supplement: Supplementary Information — Supplementary Figures 1-13, Supplementary Tables 1-5, Supplementary Notes 1-9 and Supplementary References [file ncomms13742-s1.pdf]

Supplementary Figures

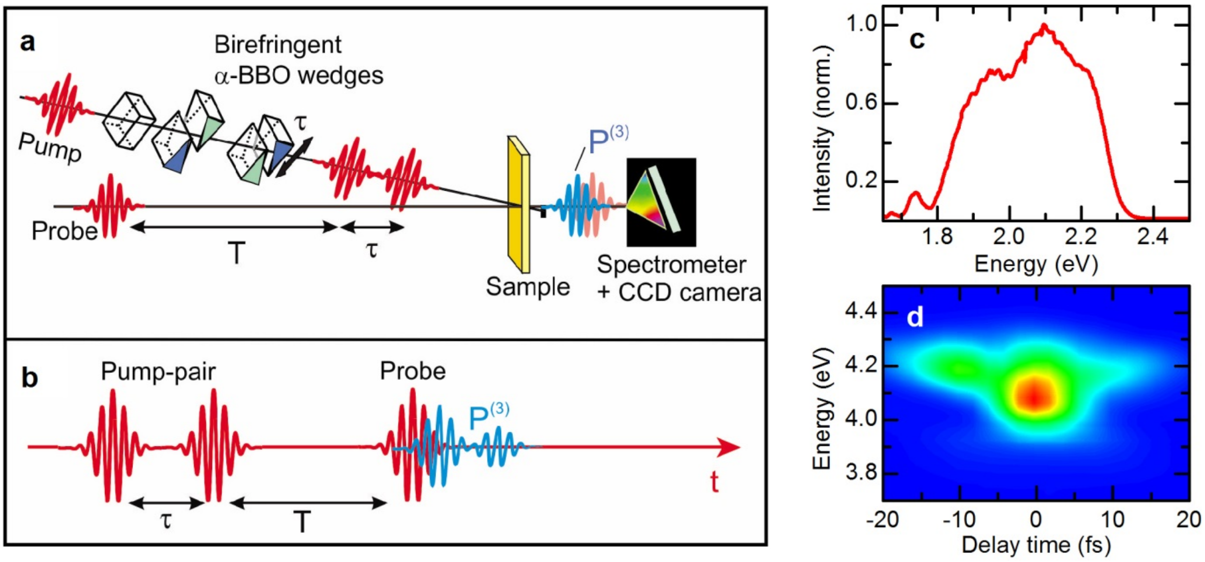

Supplementary Figure 1. Experimental setup. a, Scheme of the partially collinear 2D setup. **b,** Schematic of the pulse sequence. **c,** Intensity spectrum of the pump and probe pulse used in the measurements. **d,** SHG-FROG trace of the pump pulses, generated in a broadband NOPA.

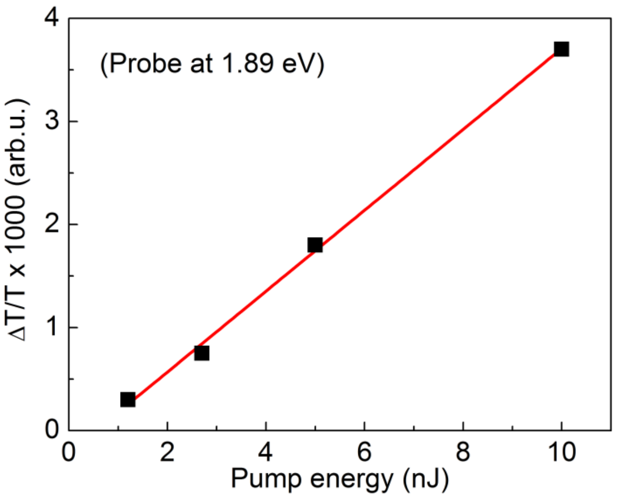

Supplementary Figure 2. Pump energy dependence of the differential transmission at the polaron pair band at probe energy of 1.89 eV. The signal is time-integrated over delay times of 600 to 800 fs. The red line is a linear fit to the experimental data (black squares). The probe pulse energy was kept at 1.5 nJ during all the measurements.

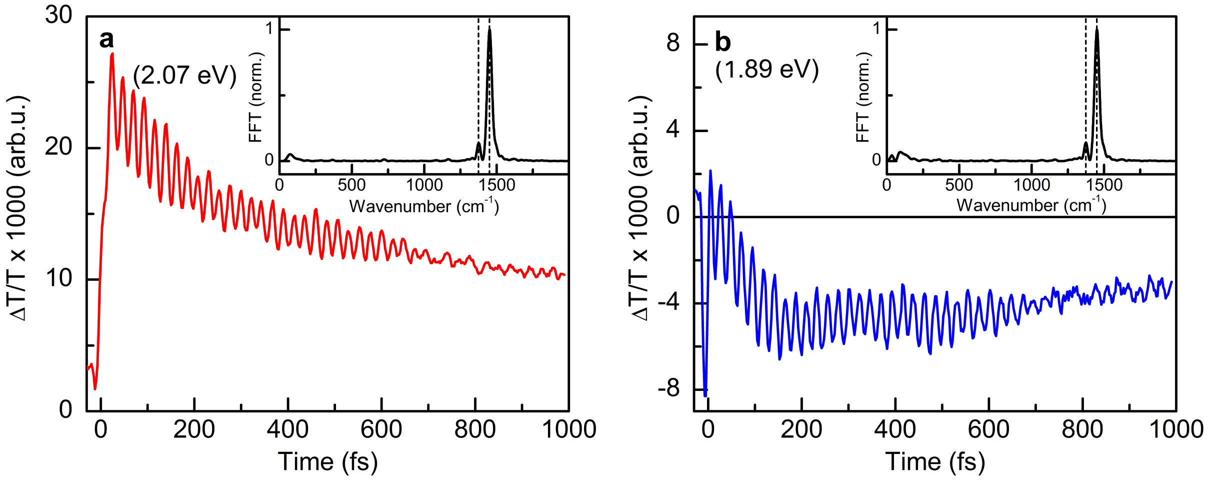

Supplementary Figure 3. Differential transmission dynamics. Differential transmission traces of **(a)** the ground state bleaching signal at a probe energy of 2.07 eV and **(b)** the excited state absorption at 1.89 eV (polaron pair band). Both dynamics show long-lived, pronounced temporal oscillations at 1450 cm^{-1} (C=C stretching mode) and a weaker mode at 1380 cm^{-1} (C-C stretching mode). The decay time of the C=C stretching vibration deduced from this measurement is about 830 fs.

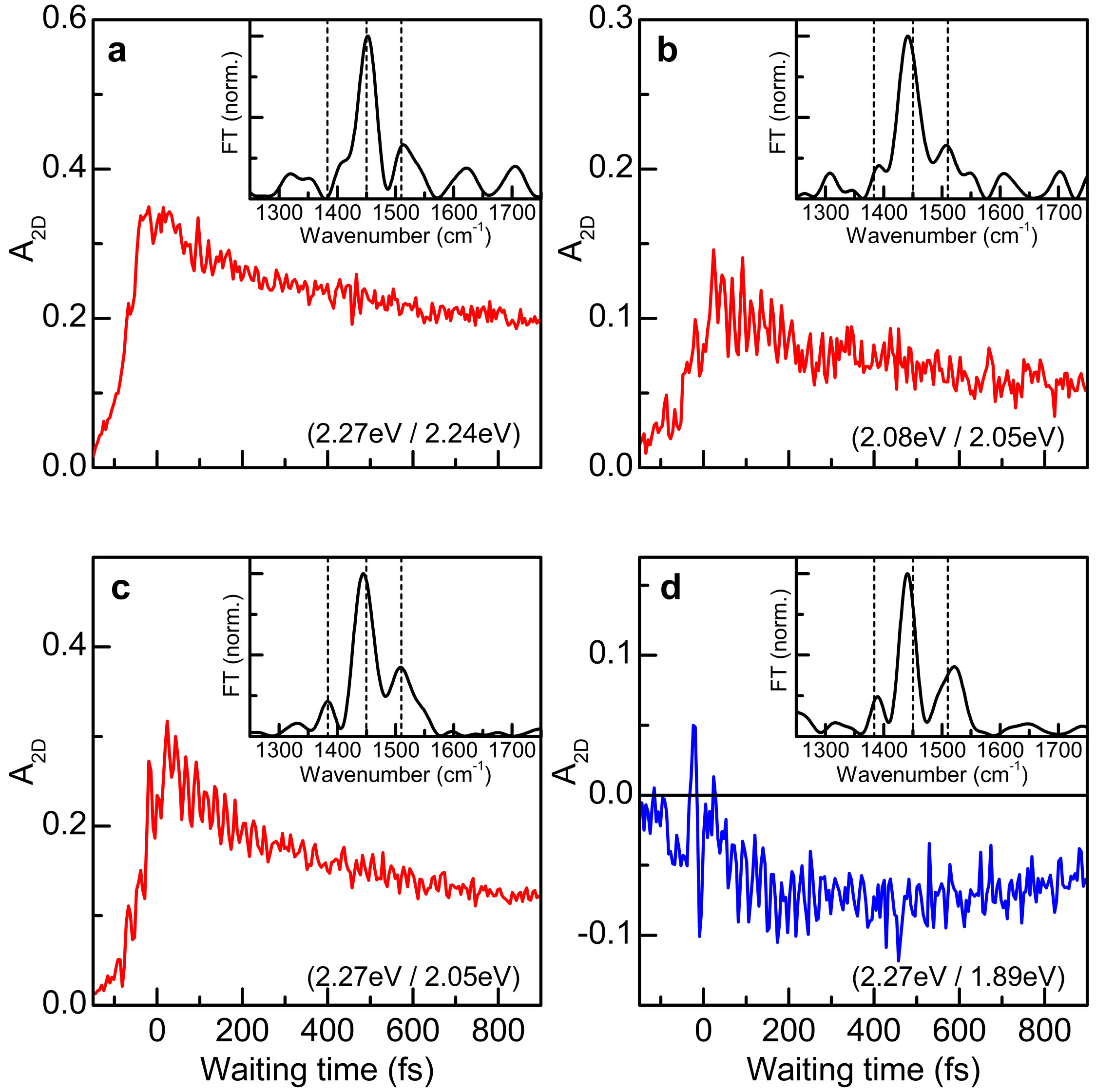

Supplementary Figure 4. Dynamics of selected peaks in the 2DES maps for waiting times of up to 900 fs. (a-b) Positive diagonal and **(c)** positive cross peaks are depicted in red. **(d)** The negative cross peak associated with photoinduced $|XP\rangle \rightarrow |P*\rangle$ absorption at a detection energy of 1.89 eV is depicted in blue. All peaks show long-lived oscillations with a dominant mode at 1450 cm^{-1} and two weaker ones at 1380 cm^{-1} and 1520 cm^{-1}, as can be seen from the Fourier transform spectra (insets). These are typical vibrational modes of the thiophene ring.

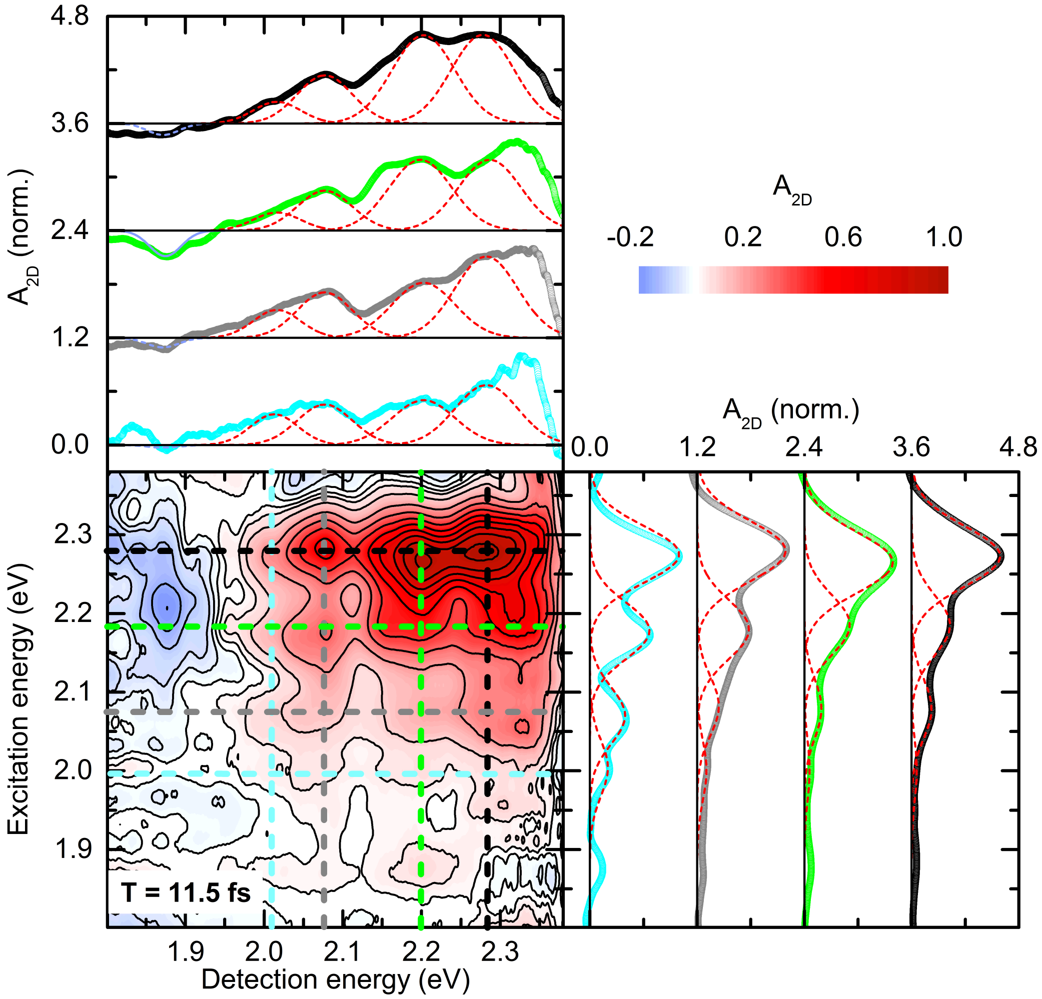

Supplementary Figure 5. 2DES map at waiting time of 11.5 fs. The cross sections along detection and excitation energy axes at each peak position are normalized to the maximum for comparison. Dashed lines on the maps show the position of the cross cuts.

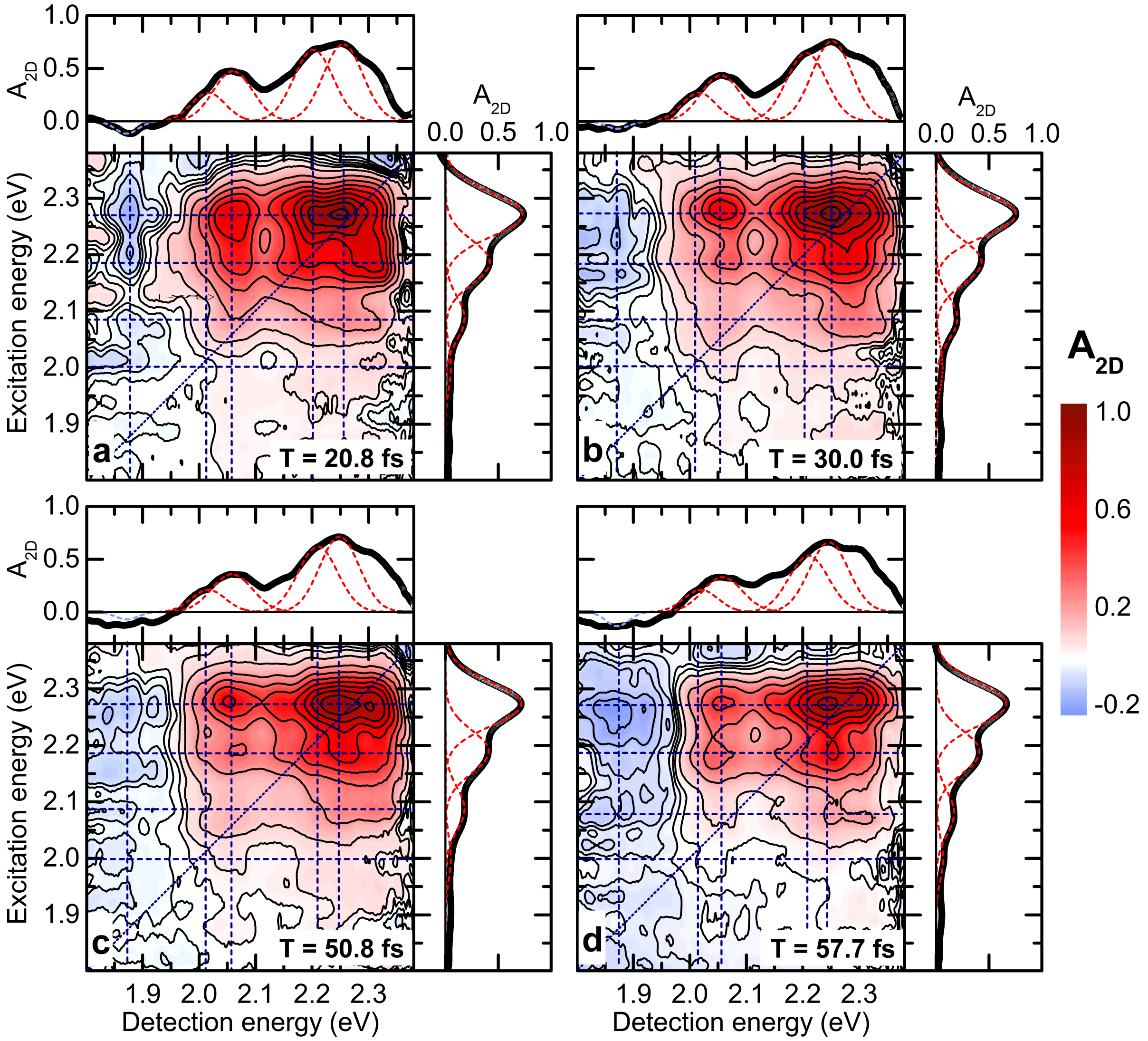

Supplementary Figure 6. 2DES maps at waiting times of 20.8 fs, 30 fs, 50.8 fs and 57.7 fs, along with their respective cross sections, clearly show that the splittings are present up to waiting times of about 60 fs. The splittings along the detection energy slowly smear out, whereas the ones along the excitation energy seem to be less affected.

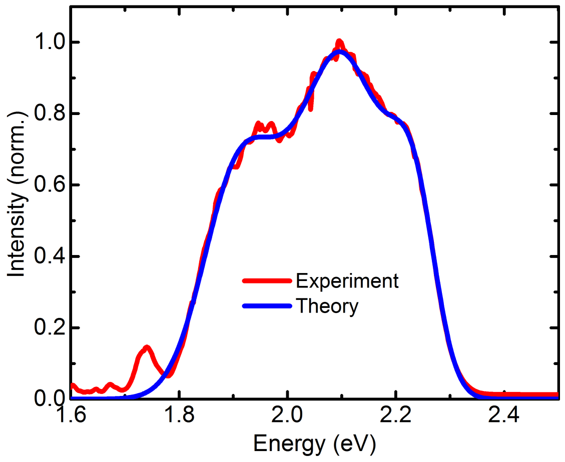

Supplementary Figure 7. Laser spectrum. The experimentally measured laser spectrum is shown in red, while the modeled laser spectrum used in 2D simulations is shown in blue. The modeled laser spectrum leads to an overall pulse duration of 7 fs of the electric field.

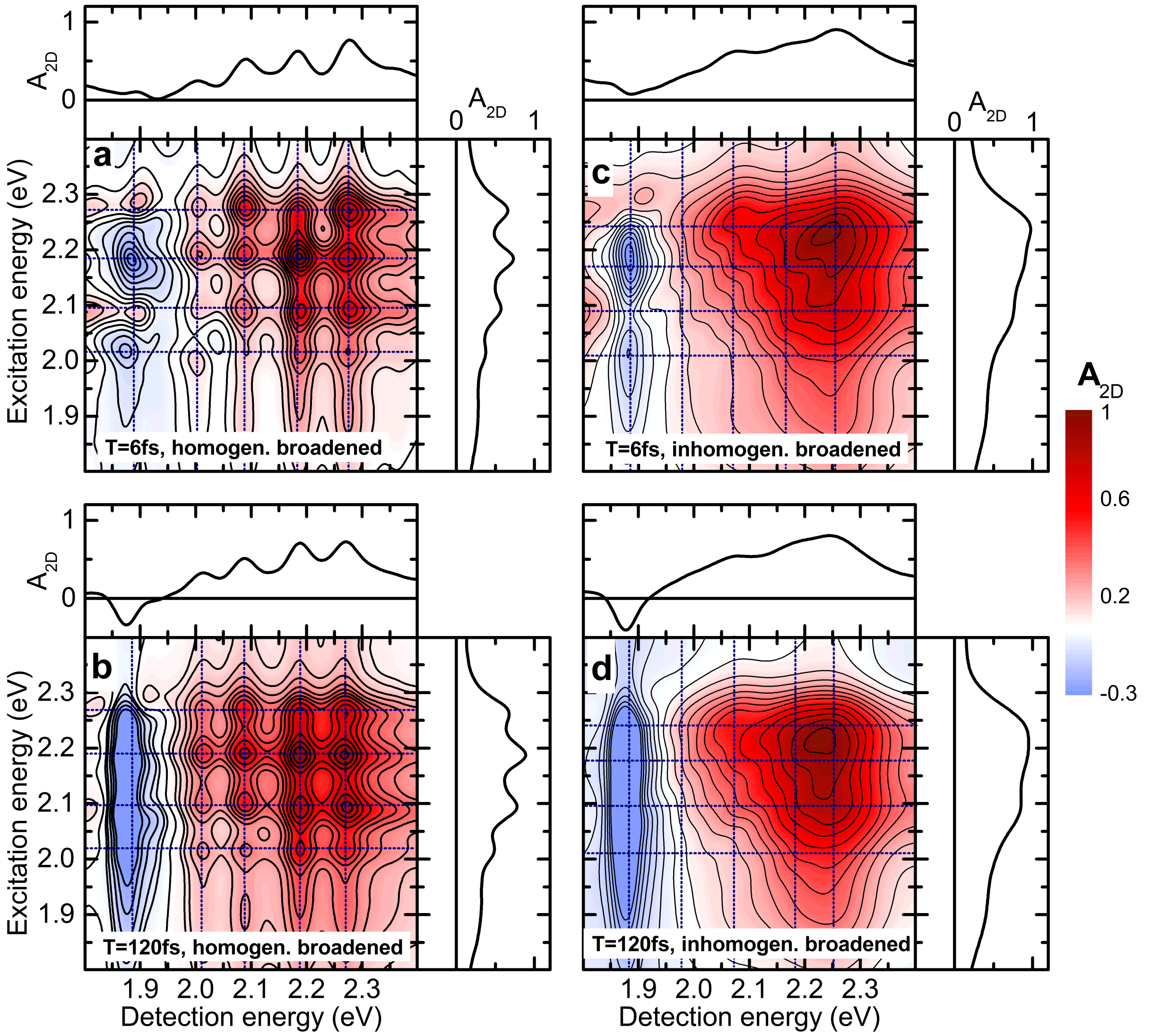

Supplementary Figure 8. Coherent model with pure dephasing and relaxation (cf. Fig. 3 in main text). Here we consider coherent polaron pair rise process in the presence of pure dephasing noise and relaxation of exciton population to polaron pair (see the parameters summarized in Supplementary Table 2). **a,** Absorptive 2D spectra at waiting time $T = 6$ fs in the absence of inhomogeneous broadening. **b,** Absorptive 2D spectra at $T = 120$ fs in the absence of inhomogeneous broadening. **c,** Absorptive 2D spectra at $T = 6$ fs in the presence of inhomogeneous broadening. **d,** Absorptive 2D spectra at $T = 120$ fs in the presence of inhomogeneous broadening.

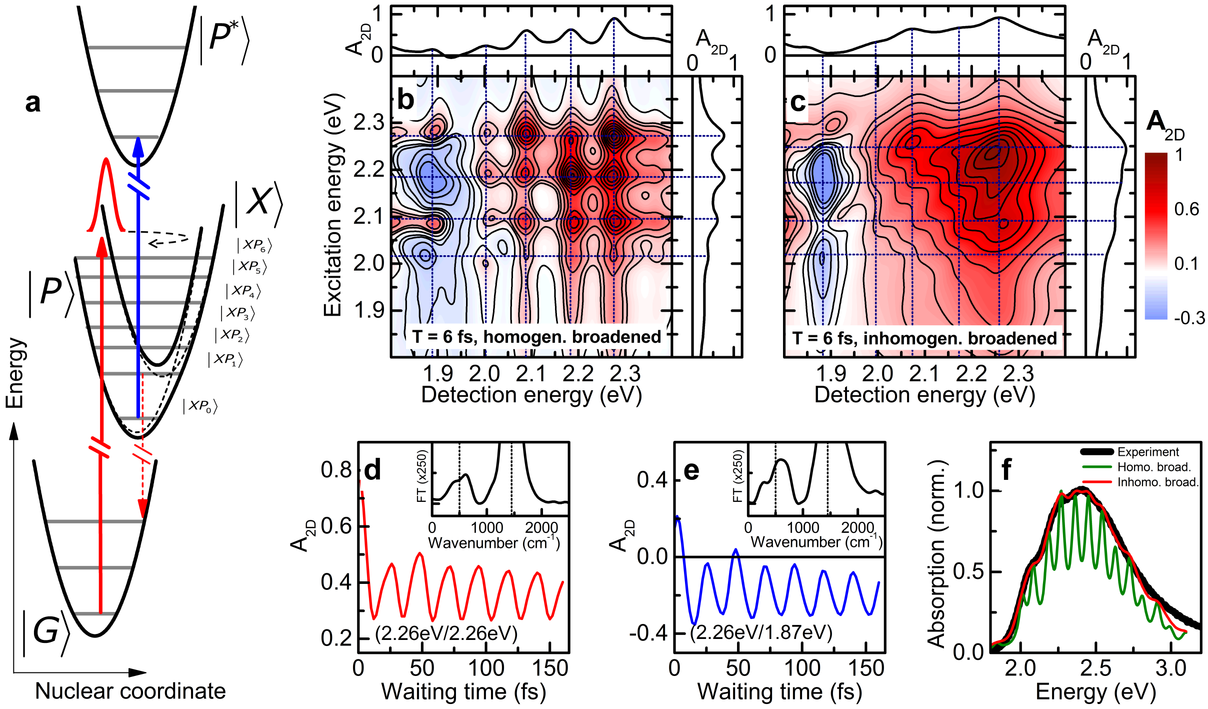

Supplementary Figure 9. Coherent model in the absence of relaxation. Here we consider coherent polaron pair rise process in the presence of pure dephasing noise only (see the parameters summarized in Supplementary Table 3). **a,** The vibronic energy levels. **b,** Absorptive 2D spectra at $T = 6$ fs in the absence of inhomogeneous broadening. **c,** Absorptive 2D spectra at $T = 6$ fs in the presence of inhomogeneous broadening. **d,** Transient at excitation energy $E_X = 2.26$ eV and detection energy $E_D = 2.26$ eV. **e,** Transient at $(E_X, E_D) = (2.26\text{ eV}, 1.87\text{ eV})$. **f,** Experimentally measured absorption spectrum (black) and simulated absorption spectra in the presence (red) and absence (green) of inhomogeneous broadening.

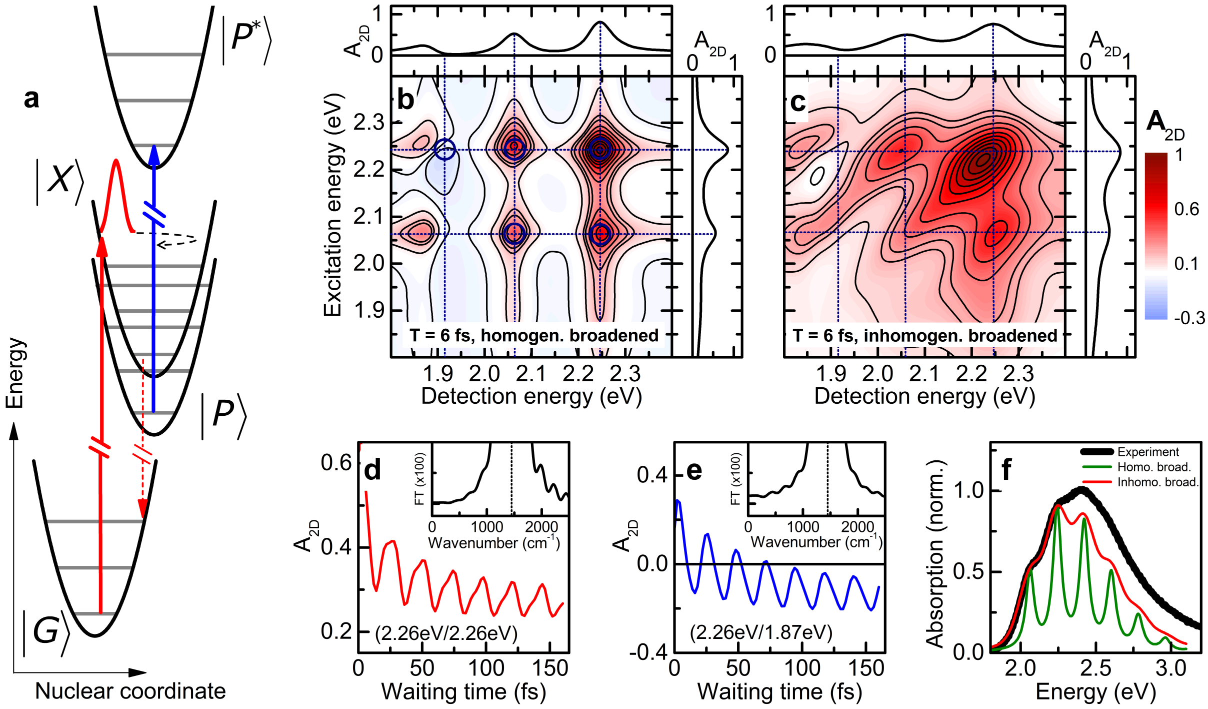

Supplementary Figure 10. Incoherent model. Here we consider incoherent polaron pair rise process in the absence of coherent electronic and vibronic couplings (see the parameters summarized in Supplementary Table 4). **a,** The vibronic energy levels. **b,** Absorptive 2D spectra at $T = 6\ \text{fs}$ in the absence of inhomogeneous broadening. **c,** Absorptive 2D spectra at $T = 6\ \text{fs}$ in the presence of inhomogeneous broadening. **d,** Transient at excitation energy $E_X = 2.26\ \text{eV}$ and detection energy $E_D = 2.26\ \text{eV}$. **e,** Transient at $(E_X, E_D) = (2.26\ \text{eV}, 1.87\ \text{eV})$. **f,** Experimentally measured absorption spectrum (black), simulated absorption spectra in the presence (red) and absence (green) of inhomogeneous broadening.

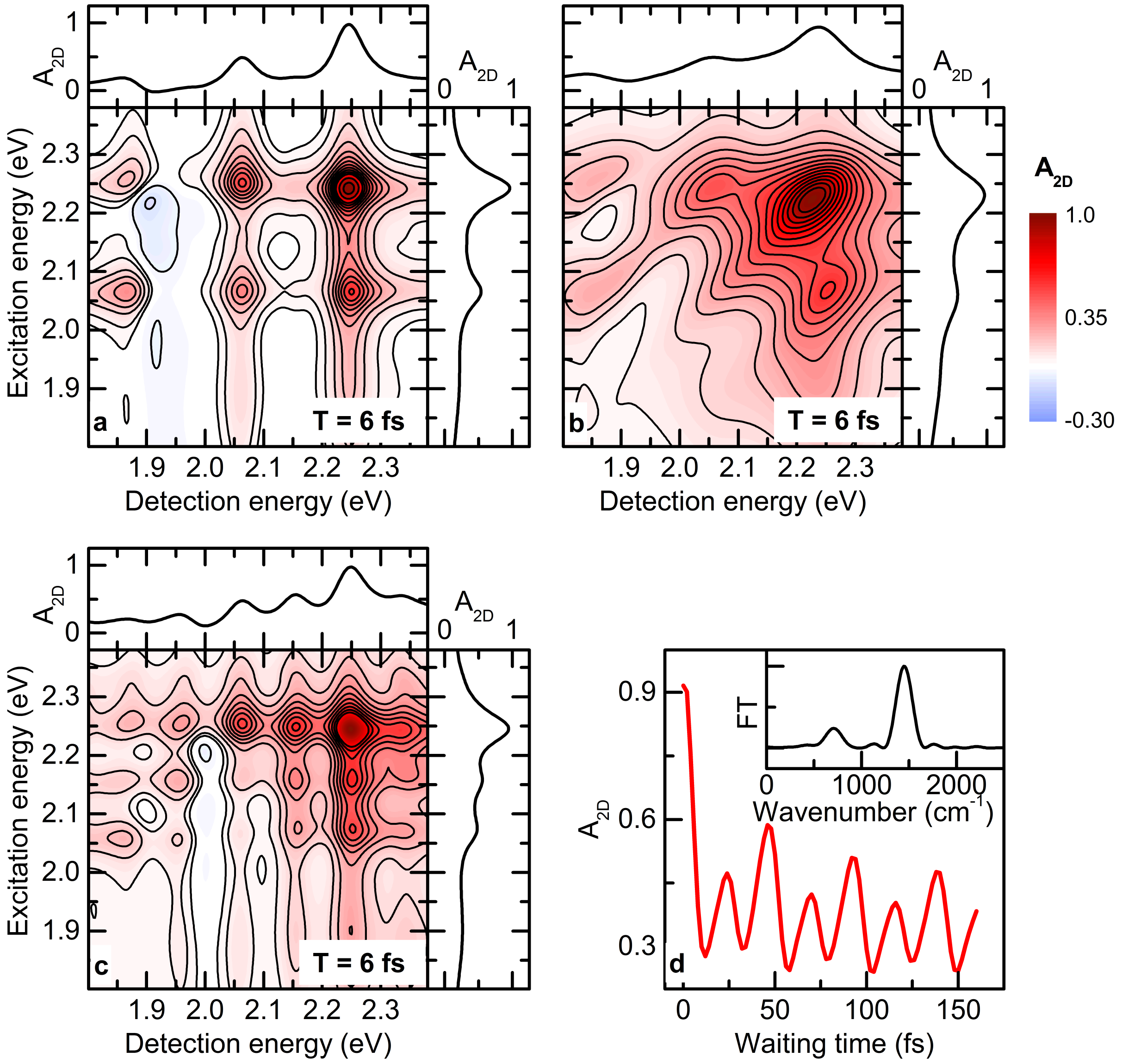

Supplementary Figure 11. Simulation of the 2DES maps and dynamics with two vibrational modes coupled to the electronic states. Electronic coupling between X and P states is assumed in the weak coupling limit. **a**, 2DES map at a waiting time of 6 fs without taking into account inhomogeneous broadening. The Huang-Rhys factor of the 730 cm^{-1} mode is of 0.05, as estimated from Fourier transform analysis of our experimental data. **b**, as in a but with inhomogeneous broadening of about 90 meV. **c**, 2DES map at a waiting time of 6 fs without inhomogeneous broadening when the Huang-Rhys factor of the 730 cm^{-1} mode is 1.5. **d**, dynamics of the exciton diagonal peak at 2.3 eV in panel c showing strong beatings of the 730 cm^{-1} and 1450 cm^{-1} modes.

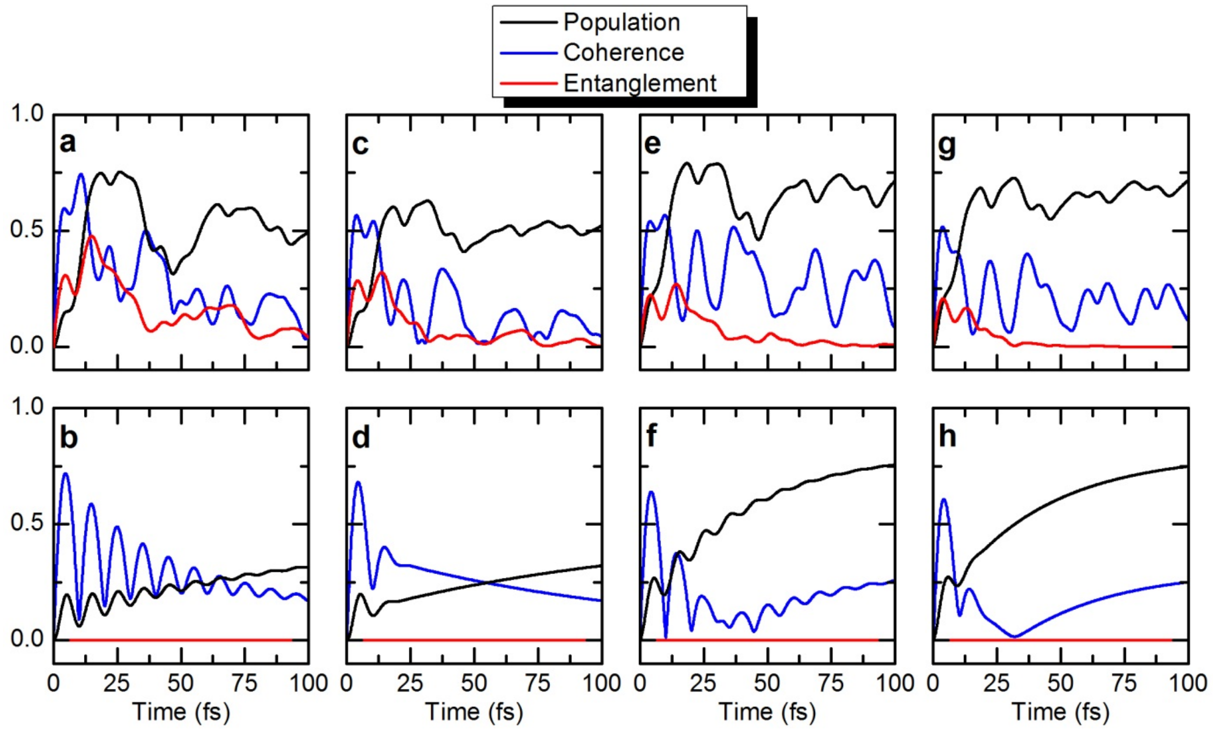

Supplementary Figure 12. The role of non-equilibrium vibrational motion in polaron pair rise dynamics. a, The time evolution of the polaron pair population (black), electronic coherence between exciton and polaron pair (blue), and the entanglement between electronic and vibrational degrees of freedom (red). Here we consider the case that non-equilibrium vibrational motion is governed by the model Hamiltonian, but the relaxation of exciton population to polaron pair is absent (cf. Supplementary Table 3). **b,** The polaron pair dynamics when the vibrational motion of the C=C stretch mode is not allowed in simulations. **c,d,** Here we consider the parameters used in (a,b), but the energy-level difference between exciton and polaron pair is modeled by a Gaussian distribution with a FWHM of 200 meV. **e-h,** Here we consider the parameters used in (a-d), but additionally consider the relaxation of excitation population to polaron pair (cf. Supplementary Table 2).

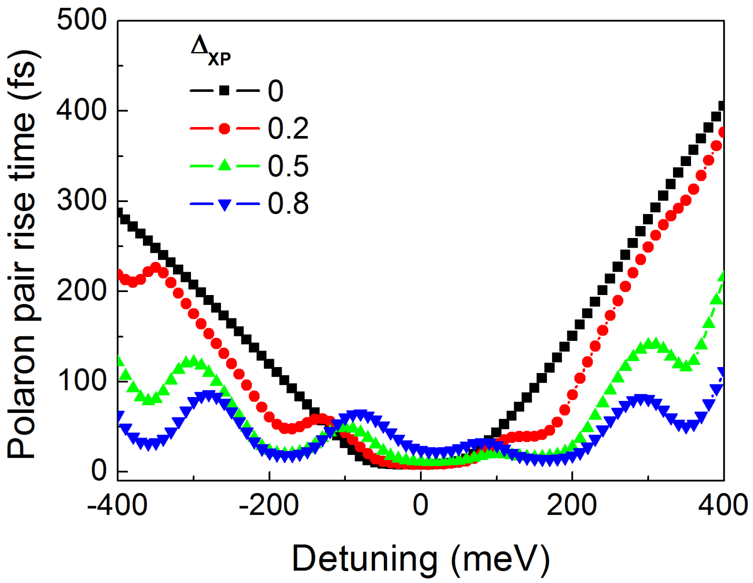

Supplementary Figure 13. Rise time of the polaron pair population as a function of the energetic detuning relative to the exciton state. The relative displacement between exciton and polaron pair potential energy surfaces, Δ_{XP}, accounts for different vibronic coupling strengths.

Supplementary Tables

k	A_k [a.u.]	υ_k [eV]	τ_k [fs]
1	0.140	2.246	21.3
2	0.488	2.108	11.8
3	0.372	1.902	12.4

Supplementary Table 1. The parameters of the electric field model.

Ω_X	2.077 eV	S_X	2.1481	ω	1450 cm^{-1}
Ω_P	1.847 eV	S_P	1.3784	γ_{dep}	1/(25 fs)
Ω_{P*}	3.711 eV	S_{P*}	1.3784	γ_{rxn}	1/(50 fs)
J	91.61 meV	S_{XP}	0.0265	Δ_{IB}	87.6 meV

Supplementary Table 2. Coherent model with pure dephasing and relaxation. A summary of model parameters used in Fig. 3 of the main text and Supplementary Figure 8.

Ω_X	2.077 eV	S_X	2.1481	ω	1450 cm^{-1}
Ω_P	1.847 eV	S_P	1.3784	γ_{dep}	1/(25 fs)
Ω_{P*}	3.711 eV	S_{P*}	1.3784	γ_{rxn}	0
J	91.61 meV	S_{XP}	0.0265	Δ_{IB}	87.6 meV

Supplementary Table 3. Coherent model in the absence of relaxation. A summary of model parameters used in Supplementary Figure 9.

Ω_X	2.063 eV	S_X	1.85	ω	1450 cm^{-1}
Ω_P	1.833 eV	S_P	1.85	γ_{dep}	1/(25 fs)
Ω_{P*}	3.697 eV	S_{P*}	1.85	γ_{rxn}	1/(50 fs)
J	0	S_{XP}	0	Δ_{IB}	146 meV

Supplementary Table 4. Incoherent model. A summary of model parameters used in Supplementary Figure 10.

$\lvert XP_k\rangle$		$\lvert XP_1\rangle$		$\lvert XP_2\rangle$		$\lvert XP_3\rangle$		$\lvert XP_4\rangle$	
Φ_k		2.01 eV		2.08 eV		2.19 eV		2.27 eV	
$\psi^{(k)}_{X,0}$	$\psi^{(k)}_{P,0}$	-0.337	-0.044	0.933	0.044	-0.110	-0.043	-0.011	0.065
$\psi^{(k)}_{X,1}$	$\psi^{(k)}_{P,1}$	-0.042	0.934	-0.054	0.330	-0.388	-0.069	0.895	0.024
$\psi^{(k)}_{X,2}$	$\psi^{(k)}_{P,2}$	-0.096	0.004	-0.045	0.113	-0.024	0.904	-0.083	0.379
$\psi^{(k)}_{X,3}$	$\psi^{(k)}_{P,3}$	-0.032	0.013	-0.024	-0.004	-0.105	-0.010	-0.062	0.196
$\psi^{(k)}_{X,4}$	$\psi^{(k)}_{P,4}$	-0.008	-0.001	-0.007	0.002	-0.041	0.019	-0.042	-0.010
$\psi^{(k)}_{X,5}$	$\psi^{(k)}_{P,5}$	-0.002	0.000	-0.002	0.000	-0.011	-0.003	-0.014	0.004
$\psi^{(k)}_{X,6}$	$\psi^{(k)}_{P,6}$	0.000	0.000	0.000	0.000	-0.003	0.000	-0.004	-0.001
$\psi^{(k)}_{X,7}$	$\psi^{(k)}_{P,7}$	0.000	0.000	0.000	0.000	0.000	0.000	-0.001	0.000
$\psi^{(k)}_{X,8}$	$\psi^{(k)}_{P,8}$	0.000	0.000	0.000	0.000	0.000	0.000	0.000	0.000
Von Neumann Entropy		0.541		0.523		0.640		0.644	
Var(σ_z)		0.439		0.434		0.581		0.608	

Supplementary Table 5. Vibronic eigenstates of the Hamiltonian. The energy levels and the amplitudes of the vibronic eigenstates in the displaced basis are displayed for the model parameters in Supplementary Table 2. For each vibronic eigenstate, the entanglement between electronic and vibrational degrees of freedom as well as the coherent mixing of exciton and polaron pair states are shown. The first two dominant amplitudes of each vibronic state in the displaced basis are marked in red.

Supplementary Notes

1. Power dependence of the transient absorption spectra. To verify that our nonlinear measurements are performed in the perturbative third-order regime and that the finite value of the pump energy does not affect the nonlinear dynamics, we have recorded and analyzed the pump energy dependence of the differential transmission at the polaron pair band (1.89 eV, Supplementary Figure 2). Pumping the sample with the same 8-fs short pulses used for the 2D measurements, we have varied the excitation energy from 1.2 to 10 nJ. The pump pulses are focused to a spot size of 50 μm (diameter) on the sample. The corresponding fluence ranges from 0.5 to 5 J m^{-2}. In the entire investigated pump energy range, the transient absorption signal at 1.89 eV (Supplementary Figure 2, black squares) scales linearly with the laser fluence. The measurements reported in the main manuscript were carried out with pump energies of about 5 nJ. The probe pulse energy was 1.5 nJ during all the measurements. This ensures a good signal-to-noise ratio while working in the third-order perturbative regime.

2. Transient absorption dynamics of rr-P3HT thin films. To compare our results with existing studies in the literature[1, 2, 3, 4, 5], transient absorption spectra of rr-P3HT thin films at room temperature were measured with the experimental setup described above. A single excitation pulse is generated by fixing the coherence time τ to zero throughout the entire measurement. The pulse energy at the sample position was adjusted to 5 nJ for the pump and 1.5 nJ for the probe, respectively. Differential transmission spectra ΔT were measured by scanning the waiting time T from -50 fs to 1 ps, with stepsize of 5 fs. The spectra were obtained by averaging a total of 5 scans for all waiting times. The transient absorption spectra at a waiting time delay of 350 fs are shown in the main text (Fig. 1b). In this section we report the time dynamics of the ground state bleaching band ($\Delta T > 0$) at a probe energy of 2.07 eV (Supplementary Figure 3a) and of the photoinduced absorption ($\Delta T < 0$) at 1.89 eV

(Supplementary Figure 3b) for time delays up to 1 ps. These detection energies correspond to the $v = 0$ exciton band (2.07 eV) and the polaron pair band (1.89 eV) in the conventional excitonic absorption model of P3HT[6]. The ground state bleaching signal at 2.07 eV is well described by a biexponential decay with a fast time constant of 100 fs and a slower one of about 7 ps. The polaron pair photoinduced absorption band at 1.89 eV shows a fast exponential rise of about 100 fs followed by a slower decay on a time scale of about 3 ps. For both probe energies, the dynamics show clear, long-lasting oscillations with a period of about 23 fs superimposed on the non-oscillatory background. These fast temporal oscillations can be described, in both cases, by an exponentially damped cosine function with a period of 23 fs and decay time of 650 fs. We take this 650 fs decay as the relaxation time of the vibrational wavepacket motion. Fourier transform intensity spectra (Supplementary Figure 3, insets) reveal the symmetric C=C stretching mode, dominating the spectrum at 1450 cm^{-1}, and a weaker feature at 1380 cm^{-1}, the symmetric C-C stretching mode. The linewidth of these Fourier transform spectra indicates a lifetime of the C=C stretching mode of 830 fs. Within the signal-to-noise ratio of our measurements, we could not detect any other oscillating component up to 2000 cm^{-1} in the transient absorption dynamics.

3. Dynamics of two-dimensional electronic spectra of rr-P3HT thin films for waiting times up to 1 ps. To study the persistence of vibronic coherence on longer timescales, we measured the dynamics of absorptive two-dimensional optical spectra for waiting times of up to 900 fs with a stepsize of 5 fs. In the measurements, the maximum energy of the pump pulse pair was set to 5 nJ at $\tau = 0$ fs, whereas the probe energy was 1.5 nJ. The dynamics of selected peaks of the 2D spectra are shown in Supplementary Figure 4 together with their Fourier transform intensity spectra (insets). The dynamics of positive diagonal (Supplementary Figure 4a, b) and cross peaks (Supplementary Figure 4c) are shown in red,

whereas the negative cross peak at $E_{\mathrm{X}} = 2.27\,\mathrm{eV}$ and $E_{\mathrm{D}} = 1.89$ eV (Supplementary Figure 4d), associated with photoinduced $|\mathrm{XP}\rangle \rightarrow |\mathrm{P}^*\rangle$ absorption (see Fig. 3a of the main text), is depicted in blue. All the positive peaks show biexponential decay dynamics with a fast time constant of about 100 fs and a slower one of several ps, similar to the dynamics observed in the differential transmission measurements (Supplementary Figure 3a). Remarkably, the negative cross peak at $E_{\mathrm{D}} = 1.89$ eV builds up during the first 100 fs and persists for about 1 ps. Moreover, all the peaks show a fast oscillatory modulation with a dominant frequency of about 1450 cm^{-1} (symmetric C=C stretch mode). The Fourier transform spectrum of each trace displays two additional peaks around 1380 cm^{-1} and 1520 cm^{-1} (Supplementary Figure 4 a-d insets, dashed lines). The peak at 1380 cm^{-1} corresponds to the symmetric C-C stretching mode and is also seen in the differential transmission measurements in Supplementary Figure 3. The peak at 1520 cm^{-1} may be assigned to the anti-symmetric C=C stretching mode of thiophene[7]. Due to the larger stepsize along the waiting time axis, the signal-to-noise ratio in these measurements is reduced in comparison to those shown in Fig. 1 of the main text.

4. Details of the experimental spectral splittings up to 60 fs

To better show that each of the exciton peaks predicted by the incoherent model[6] is indeed split into a quartet, here we show a 2DES map at waiting time of 11.5 fs along with the cross sections, not only around 2.25 eV, but at each peak position for both the excitation and detection energy axes. The quartet structures are clearly visible at all peak positions, as depicted in Supplementary Figure 5.

Additionally, to show that the subpeak structure is indeed visible up to about 60 fs, we also report 2DES maps at later waiting times (Supplementary Figure 6) with their respective cross sections at 2.25 eV. From these maps and cross sections one can clearly recognize that the peak splitting along the detection energy persists for up to $T = 58$ fs.

5. Theoretical model

The Hamiltonian. To analyze the experimentally observed linear absorption and 2DES spectra and to understand the basic physical mechanisms underpinning these observations, we consider a phenomenological model for P3HT consisting of four electronic states (ground state, exciton, polaron pair and excited polaron pair) coupled to a vibrational degree of freedom provided by the C=C stretch mode. This reduced model is described by the Hamiltonian of the form

$$\begin{aligned} \mathrm{H} = &|\mathrm{G}\rangle\langle\mathrm{G}|(\hbar\omega \mathrm{a}^{\dagger}\mathrm{a}) + |\mathrm{X}\rangle\langle\mathrm{X}|(\hbar\Omega_{\mathrm{X}} + \hbar\omega \mathrm{a}^{\dagger}\mathrm{a} + \hbar\omega\sqrt{S_{\mathrm{X}}}(\mathrm{a}^{\dagger}+\mathrm{a}) + \hbar\omega S_{\mathrm{X}}) \\ &+ |\mathrm{P}\rangle\langle\mathrm{P}|(\hbar\Omega_{\mathrm{P}} + \hbar\omega \mathrm{a}^{\dagger}\mathrm{a} + \hbar\omega\sqrt{S_{\mathrm{P}}}(\mathrm{a}^{\dagger}+\mathrm{a}) + \hbar\omega S_{\mathrm{P}}) \\ &+ (|\mathrm{X}\rangle\langle\mathrm{P}| + |\mathrm{P}\rangle\langle\mathrm{X}|)(\hbar J + \hbar\omega\sqrt{S_{\mathrm{XP}}}(\mathrm{a}^{\dagger}+\mathrm{a})) \\ &+ |\mathrm{P}*\rangle\langle\mathrm{P}*|(\hbar\Omega_{\mathrm{P}*} + \hbar\omega \mathrm{a}^{\dagger}\mathrm{a} + \hbar\omega\sqrt{S_{\mathrm{P}*}}(\mathrm{a}^{\dagger}+\mathrm{a}) + \hbar\omega S_{\mathrm{P}*}). \end{aligned} \quad (2)$$

Here $|\mathrm{G}\rangle, |\mathrm{X}\rangle, |\mathrm{P}\rangle, |\mathrm{P}*\rangle$ denote ground state, exciton, polaron pair and excited polaron pair state, respectively, with the purely electronic energies represented by $\Omega_{\mathrm{X}}, \Omega_{\mathrm{P}}, \Omega_{\mathrm{P}*}$. The site energy of each electronic state is given by the sum of the purely electronic energy Ω_{k} and the reorganization energy ωS_{k} due to the vibronic coupling. The electronic coupling between exciton and polaron pair denoted by J is responsible for the coherent population transfer between exciton and polaron pair. The C=C stretch mode is modeled by an effective, single vibrational mode (annihilation and creation operators denoted by a and $\mathrm{a}^{\dagger}$, respectively) with vibrational frequency of $\hbar\omega = 1450\,\mathrm{cm}^{-1}$ and a vibrational relaxation rate of $\gamma_{\mathrm{vib}} = (650\,\mathrm{fs})^{-1}$, both of which are determined based on experimentally observed oscillatory pump-probe and 2D signals. The interaction between electronic states and the C=C stretch mode is modeled by vibronic couplings quantified by Huang-Rhys (HR) factors that we denote as $S_{\mathrm{X}}, S_{\mathrm{P}}, S_{\mathrm{P}*}, S_{\mathrm{XP}}$. Here $S_{\mathrm{X}}, S_{\mathrm{P}}, S_{\mathrm{P}*}$ describe the displacement of the equilibrium position of the vibrational mode

in the electronic excited states with respect to the equilibrium position of the mode in the electronic ground state. These three vibronic couplings describe the modulation of the excited state energy $\hbar\tilde{\Omega}_{\mathrm{k}} = \hbar\Omega_{\mathrm{k}} + \hbar\omega a^{\dagger}a + \hbar\omega\sqrt{S_{\mathrm{k}}}(a^{\dagger} + a) + \hbar\omega S_{\mathrm{k}}$ (cf. $\mathrm{k} = \mathrm{X}, \mathrm{P}, \mathrm{P}^*$) due to the vibrational motion. It is notable that when $S_{\mathrm{X}} \neq S_{\mathrm{P}}$, the energy-level difference $\tilde{\Omega}_{\mathrm{X}} - \tilde{\Omega}_{\mathrm{P}}$ between exciton and polaron pair states depends on $\omega(\sqrt{S_{\mathrm{X}}} - \sqrt{S_{\mathrm{P}}})(a^{\dagger} + a)$, implying that non-equilibrium vibrational motion can dynamically modulate the resonance between exciton and polaron pair states significantly when the difference in the corresponding HR factors is sufficiently large[8]. On the other hand, S_{XP} describes the modulation of the electronic coupling strength between exciton and polaron pair due to the vibrational motion. In the case of natural photosynthetic systems, it has been shown that typically the vibronic modulation of the energy levels leads to larger HR factors than that modulating electronic couplings[9]. In simulations, we found that larger $S_{\mathrm{X}}, S_{\mathrm{P}}, S_{\mathrm{P}*}$ than S_{XP} are needed to reproduce experimental observations within our model.

Decoherence. The unavoidable coupling of the electronic states to their environments destroys electronic coherences and induces homogeneous broadening and incoherent features in the population transfer dynamics between excited states. In the absence of a detailed microscopic model for the dephasing of vibronic excitations in the polymer, the influence of the noisy environment on electronic states can be modeled phenomenologically by dissipative (i.e. non-unitary) contributions to the time evolution of the vibronic system that we assume can be expressed in the so-called Lindblad form:

$$L_{\mathrm{dep}}[\rho(t)] = \sum_{\mathrm{k=G,X,P,P^*}} \gamma_{\mathrm{dep}} \left(|\mathrm{k}\rangle\langle \mathrm{k}|\rho(t)|\mathrm{k}\rangle\langle \mathrm{k}| - \frac{1}{2}\{|\mathrm{k}\rangle\langle \mathrm{k}|, \rho(t)\} \right), \tag{3}$$

$$L_{\mathrm{rxn}}[\rho(t)] = \gamma_{\mathrm{rxn}} \left(|\mathrm{P}\rangle\langle \mathrm{X}|\rho(t)|\mathrm{X}\rangle\langle \mathrm{P}| - \frac{1}{2}\{|\mathrm{X}\rangle\langle \mathrm{X}|, \rho(t)\} \right). \tag{4}$$

Here the first Lindblad super-operator describes pure dephasing noise with a rate of γ_{dep}, which contributes to homogeneous broadening but does not induce population transfer between exciton and polaron pair states by itself. The second Lindblad super-operator describes the incoherent population relaxation from the exciton to the polaron pair state with a rate of γ_{rxn}. The vibrational relaxation of the C=C stretch mode is modeled by a formally analogous term

$$L_{\mathrm{vib}}[\rho(t)] = \gamma_{\mathrm{vib}} \left(2\tilde{a}\rho(t)\tilde{a}^\dagger - \{\tilde{a}^\dagger\tilde{a}, \rho(t)\} \right), \tag{5}$$

where the relaxation rate of the vibration is taken to be $\gamma_{\mathrm{vib}} = (650\ \mathrm{fs})^{-1}$ based on experimental results. To take into account the fact that the equilibrium position of the vibrational mode depends on the electronic states, we introduce a displaced mode operator $\tilde{a} = U^\dagger a U$ with a unitary operator $U^\dagger = |\mathrm{G}\rangle\langle \mathrm{G}| \otimes D^\dagger(0) + \sum_{\mathrm{k=X,P,P^*}} |\mathrm{k}\rangle\langle \mathrm{k}| \otimes D^\dagger(\sqrt{S_{\mathrm{k}}})$ in terms of the so-called displacement operator $D(x) = \exp(x(a^\dagger - a))$. The Lindblad super-operator in terms of $\tilde{a}$ therefore describes the relaxation of the vibrational motion to different equilibrium positions depending on the electronic state of the system. When the HR factors are sufficiently small, $\tilde{a}$ is well approximated by a, leading to a widely used Lindblad operator for mode damping. However, due to the large HR factors of the present system, such an approximation is not valid here.

For the Hamiltonian and noise model described above, the time evolution of the global, vibronic (electronic + vibrational) state $\rho(t)$ is governed by the quantum master equation

$$\frac{d\rho(t)}{dt} = -\frac{i}{\hbar}[H, \rho(t)] + L_{dep}[\rho(t)] + L_{rxn}[\rho(t)] + L_{vib}[\rho(t)]. \quad (6)$$

We note that the above phenomenological model is employed to demonstrate how coherent electronic and vibronic couplings affect 2D optical responses in the presence of noise and disorder. We found that, remarkably, several relevant experimental features, such as multi-frequency transients and splitting of 2D peaks, can be reproduced even though the employed model is rather simple. A detailed microscopic model based on electronic wavefunctions and realistic phonon spectral densities may be able to reproduce more detailed features of experimental observations, such as the clean splitting of 2D peaks and the spectral diffusion-like effects discussed in the manuscript, and also provide a more accurate description of the transient dynamics of 2D spectra.

Absorptive 2D spectra. To calculate the absorptive 2D spectra of the present system, we consider the transition dipole between the ground and exciton state and that between the polaron pair and excited polaron pair states, denoted by $\vec{\mu}_X$ and $\vec{\mu}_P$, respectively, with the assumption that the transition dipole strength between ground state and polaron pair is sufficiently weak. In this case, the optical response from the polaron pair state can be observed mediated by the optical transition between polaron pair and excited polaron pair states, leading to excited state absorption (ESA) signals in pump probe and 2D spectra.

The third-order polarization responsible for 2D spectra[10] is described by

$$P^{(3)}(t) = \int_0^\infty dt_3 \int_0^\infty dt_2 \int_0^\infty dt_1 S^{(3)}(t_3, t_2, t_1) E(t - t_3) E(t - t_3 - t_2) E(t - t_3 - t_2 - t_1), \quad (7)$$

where the electric field $E(t)$ is modeled by a sum of three Gaussian fields to fit the laser spectrum used in experiments, as shown in Supplementary Figure 7 and Supplementary Table 1, with a carrier frequency of υ and a slowly varying, time-dependent function $\varepsilon(t)$

$$\mathrm{E}(t) = \varepsilon(t+T+\tau)\exp(-i\,\upsilon\,(t+T+\tau) + i\,\vec{\mathbf{k}}_1\cdot\vec{\mathbf{r}}) \\ + \varepsilon(t+T)\exp(-i\,\upsilon\,(t+T) + i\,\vec{\mathbf{k}}_2\cdot\vec{\mathbf{r}}) + \varepsilon(t)\exp(-i\,\upsilon\,t + i\,\vec{\mathbf{k}}_3\cdot\vec{\mathbf{r}}) + \mathrm{c.c}, \tag{8}$$

$$\varepsilon(t) = \sum_{\mathrm{k}=1}^{3} A_{\mathrm{k}} \exp(-2\ln(2)\,(t/\tau_{\mathrm{k}})^2 + i(\upsilon - \upsilon_{\mathrm{k}})t), \tag{9}$$

where c.c denotes the complex conjugate. Here τ, T, t represent the time delays between the first and second excitation pulses, between the second and third excitation pulses, and between the third excitation pulse and the emerging signal from the system, respectively. The Fourier transformation of the third-order polarization with respect to (τ, t) leads to 2D spectra as a function of excitation and detection energies $(E_{\mathrm{X}}, E_{\mathrm{D}})$ for each waiting time T.

In this work, we calculated absorptive 2D spectra within the rotating wave approximation[7] by taking into account only the signal contributions emitted into two phase-matched directions $-\vec{\mathbf{k}}_1 + \vec{\mathbf{k}}_2 + \vec{\mathbf{k}}_3$ and $\vec{\mathbf{k}}_1 - \vec{\mathbf{k}}_2 + \vec{\mathbf{k}}_3$. We note that for our model, the influence of the transition dipoles and their rotational averaging (ensemble) on the third-order optical response $\mathrm{S}^{(3)}(t_3,t_2,t_1)$ is characterized by two effective transition dipole strengths. More specifically, here we consider the ground state bleaching (GSB), stimulated emission (SE) and excited state absorption (ESA) components of the rephasing pathway, whose contributions to the third-order optical response are given by

$$\mathrm{S}^{(3)}_{\mathrm{R,GSB}}(t_3,t_2,t_1) = \mathrm{Tr}\Big[\mu^{-}_{\mathrm{X}}\mathrm{u}(t_3)[\mu^{+}_{\mathrm{X}}\mathrm{u}(t_2)[\mathrm{u}(t_1)[\rho_{\mathrm{eq}}\mu^{-}_{\mathrm{X}}]\mu^{+}_{\mathrm{X}}]]\Big], \tag{10}$$

$$\mathrm{S}^{(3)}_{\mathrm{R,SE}}(t_3,t_2,t_1) = \mathrm{Tr}\Big[\mu^{-}_{\mathrm{X}}\mathrm{u}(t_3)[\mathrm{u}(t_2)[\mu^{+}_{\mathrm{X}}\mathrm{u}(t_1)[\rho_{\mathrm{eq}}\mu^{-}_{\mathrm{X}}]]\mu^{+}_{\mathrm{X}}]\Big], \tag{11}$$

$$\mathrm{S}^{(3)}_{\mathrm{R,ESA}}(t_3,t_2,t_1) = \mathrm{Tr}\Big[\mu^{-}_{\mathrm{P}}\mathrm{u}(t_3)[\mu^{+}_{\mathrm{P}}\mathrm{u}(t_2)[\mu^{+}_{\mathrm{X}}\mathrm{u}(t_1)[\rho_{\mathrm{eq}}\mu^{-}_{\mathrm{X}}]]]\Big]. \tag{12}$$

Here ρ_{eq} denotes the equilibrium state of the vibronic system in the electronic ground state, $\mathrm{u}(t)$ is a formal expression of the time evolution operator describing both the Hamiltonian and noise, and $\mu_{X,P}^{\pm}$ are the transition dipole operators defined by

$$\mu_{\mathrm{X}}^{+} = \hat{\mathbf{e}}\cdot\vec{\boldsymbol{\mu}}_{\mathrm{X}}\left|\mathrm{X}\right\rangle\left\langle\mathrm{G}\right|, \mu_{\mathrm{X}}^{-} = \hat{\mathbf{e}}\cdot\vec{\boldsymbol{\mu}}_{\mathrm{X}}\left|\mathrm{G}\right\rangle\left\langle\mathrm{X}\right|, \mu_{\mathrm{P}}^{+} = \hat{\mathbf{e}}\cdot\vec{\boldsymbol{\mu}}_{\mathrm{P}}\left|\mathrm{P}^{*}\right\rangle\left\langle\mathrm{P}\right|, \mu_{\mathrm{P}}^{-} = \hat{\mathbf{e}}\cdot\vec{\boldsymbol{\mu}}_{\mathrm{P}}\left|\mathrm{P}\right\rangle\left\langle\mathrm{P}^{*}\right|, \quad (13)$$

with $\hat{\mathrm{e}}$ denoting the unit vector parallel to the polarization of the electric field. In this case, the third-order optical responses are reduced to

$$\mathrm{S}_{\mathrm{R,GSB}}^{(3)}(t_3,t_2,t_1) = (\hat{\mathbf{e}}\cdot\vec{\boldsymbol{\mu}}_{\mathrm{X}})^4\,\mathrm{Tr}\Big[\left|\mathrm{G}\right\rangle\left\langle\mathrm{X}\right|\mathrm{u}(t_3)[\left|\mathrm{X}\right\rangle\left\langle\mathrm{G}\right|\mathrm{u}(t_2)[\mathrm{u}(t_1)[\rho_{\mathrm{eq}}\left|\mathrm{G}\right\rangle\left\langle\mathrm{X}\right|]\left|\mathrm{X}\right\rangle\left\langle\mathrm{G}\right|]]\Big], \quad (14)$$

$$\mathrm{S}_{\mathrm{R,SE}}^{(3)}(t_3,t_2,t_1) = (\hat{\mathbf{e}}\cdot\vec{\boldsymbol{\mu}}_{\mathrm{X}})^4\,\mathrm{Tr}\Big[\left|\mathrm{G}\right\rangle\left\langle\mathrm{X}\right|\mathrm{u}(t_3)[\mathrm{u}(t_2)[\left|\mathrm{X}\right\rangle\left\langle\mathrm{G}\right|\mathrm{u}(t_1)[\rho_{\mathrm{eq}}\left|\mathrm{G}\right\rangle\left\langle\mathrm{X}\right|]]\left|\mathrm{X}\right\rangle\left\langle\mathrm{G}\right|]\Big], \quad (15)$$

$$\mathrm{S}_{\mathrm{R,ESA}}^{(3)}(t_3,t_2,t_1) = (\hat{\mathbf{e}}\cdot\vec{\boldsymbol{\mu}}_{\mathrm{X}})^2(\hat{\mathbf{e}}\cdot\vec{\boldsymbol{\mu}}_{\mathrm{P}})^2\,\mathrm{Tr}\Big[\left|\mathrm{P}\right\rangle\left\langle\mathrm{P}^{*}\right|\mathrm{u}(t_3)[\left|\mathrm{P}^{*}\right\rangle\left\langle\mathrm{P}\right|\mathrm{u}(t_2)[\left|\mathrm{X}\right\rangle\left\langle\mathrm{G}\right|\mathrm{u}(t_1)[\rho_{\mathrm{eq}}\left|\mathrm{G}\right\rangle\left\langle\mathrm{X}\right|]]]\Big]. \quad (16)$$

Therefore the rotational average of the transition dipoles (ensemble) with respect to the polarization direction of the electric field is characterized by two parameters $\mu_{\mathrm{GSB,SE}} = \left\langle(\hat{\mathbf{e}}\cdot\vec{\boldsymbol{\mu}}_{\mathrm{X}})^4\right\rangle$ and $\mu_{\mathrm{ESA}} = \left\langle(\hat{\mathbf{e}}\cdot\vec{\boldsymbol{\mu}}_{\mathrm{X}})^2(\hat{\mathbf{e}}\cdot\vec{\boldsymbol{\mu}}_{\mathrm{P}})^2\right\rangle$, where $\mu_{\mathrm{GSB,SE}}$ and μ_{ESA} determine the relative intensity between the GSB/SE contributions and ESA contribution, respectively. In this work, we take $\mu_{\mathrm{ESA}} \approx 0.2\,\mu_{\mathrm{GSB,SE}}$ to take into account the fact that the experimentally observed (negative-valued) ESA signals at detection energies around 1.89 eV are weaker than the (positive-valued) GSB/SE signals at detection energies between 2.0 eV and 2.4 eV (cf. Fig. 1 and 2 in the main text). Similarly, the non-rephasing and double coherence pathways can be characterized by these effective transition dipole strengths $\mu_{\mathrm{GSB,SE}}$ and μ_{ESA}.

Simulated absorptive 2D spectra

Model 1: Coherent polaron pair rise under pure dephasing noise and relaxation. In Fig. 3 of the manuscript, we considered a coherent model where the population transfer between exciton and polaron pair states is governed by coherent electronic and vibronic couplings as well as the relaxation of exciton population to the polaron pair state (cf. $J \neq 0, S_{XP} \neq 0, \gamma_{rxn} \neq 0$, see the model parameters summarized in Supplementary Table 2). In Supplementary Figure 8c-d, for the parameters used for Fig. 3, absorptive 2D spectra in the presence of inhomogeneous broadening are displayed. The inhomogeneous broadening was modeled by a Gaussian distribution of the exciton energy Ω_X with a FWHM of $\Delta_{IB} = 87.6$ meV where the differences in electronic energies $\Omega_X, \Omega_P, \Omega_{P*}$ are kept unchanged, and then we additionally considered the inhomogeneous broadening of the electronic energy difference $\Omega_X - \Omega_P$ between exciton and polaron pair modeled by an independent Gaussian distribution with a FWHM of 50 meV. We found that the inhomogeneous broadening of the electronic energy difference does not change significantly the overall 2D lineshapes and specifically maintains the splitting of 2D peaks for a FWHM from 0 up to ~50 meV. The robustness of the splitting against the variation of the electronic energy difference is due to the strong electronic coupling of ~92 meV and strong vibronic couplings used in the simulations (cf. Supplementary Table 2). In this parameter regime the mixing of exciton and polaron pair occurs over a wide range of the electronic energy difference between them and leads to the splitting of 2D peaks. Compared to the case where inhomogeneous broadening is absent (cf. Supplementary Figure 8a, b), the splitting of 2D peaks is less visible in the presence of inhomogeneous broadening (cf. Supplementary Figure 8c, d), but the splitting is still visible in the cross sections at both excitation and detection energy of 2.26 eV (averaged over 2.25-2.27 eV). We found that when the FWHM of the inhomogeneous broadening of the electronic energy difference $\Omega_X - \Omega_P$ becomes larger than ~50 meV, there are notable changes in

simulated absorption and 2D spectra, but we found decreasing agreement with the experimental results. We also considered an alternative inhomogeneous broadening model where each value of the electronic energies $\Omega_{\mathrm{X}}, \Omega_{\mathrm{P}}, \Omega_{\mathrm{P*}}$ is modeled by an independent Gaussian distribution, but could not find a better match between theory and experiments. We found that the experimentally observed clean splitting of 2D peaks is unlikely to be reproduced accurately within the level of the current phenomenological model if we optimize the inhomogeneous broadening such that the absorption spectrum is well reproduced. This may be due to the limitations of the noise model we used, as it also cannot describe spectral diffusion-like effects observed in experiments nor any non-Markovian behavior in the environmental coupling, but it may also depend on the inhomogeneous broadening model. Nevertheless, our simulations do support the experimental finding that the mixing between exciton and polaron pair states can induce the splitting of 2D peaks even if it is not visible in the linear absorption spectrum.

Model 2: Coherent polaron pair rise in the absence of relaxation. Now we consider the case where the relaxation rate of exciton population to polaron pair state is set to zero (cf. $J \neq 0, S_{\mathrm{XP}} \neq 0, \gamma_{\mathrm{rxn}} = 0$, see the model parameters summarized in Supplementary Table 3). As shown in Supplementary Figure 9, even in the absence of the incoherent relaxation, the splitting of 2D peaks and multi-frequency transients are obtained in simulations, implying that these features are related to the coherent electronic and vibronic couplings. However, as shown in Supplementary Figure 9e, the transient associated with the polaron pair rise looks less exponential-like, contrary to the experimental observations. This may be due to the fact that the pure dephasing noise considered here is accurate only at sufficiently high temperatures as the steady state under the pure dephasing noise does not result in a higher population at a lower energy state at least in the absence of vibronic couplings. These results suggest that the splitting of 2D peaks and multi-frequency transients are the signatures of the

coherent couplings of the present system, but a more sophisticated noise model may be required to reproduce detailed features of experimentally observed 2D spectra.

Model 3: Incoherent polaron pair rise in the absence of coherent couplings. To demonstrate that the splitting of 2D peaks and multi-frequency transients originate from coherent electronic and vibronic couplings, here we consider an incoherent model where the polaron pair rise is dominated by the relaxation process (cf. $J = 0, S_{\mathrm{XP}} = 0, \gamma_{\mathrm{rxn}} \neq 0$, see the model parameters summarized in Supplementary Table 4). As shown in Supplementary Figure 10f, in the absence of the coherent electronic and vibronic couplings, the homogeneously broadened absorptive peaks shown in green do not exhibit any splitting in contrast to Fig. 3f in the manuscript and Supplementary Figure 9f. In this case, the homogeneously broadened 2D spectra also do not exhibit any splitting, as shown in Supplementary Figure 10b where inhomogeneous broadening is not considered. Even in the presence of inhomogeneous broadening, which is optimized to fit the first two vibrational progressions in the absorption spectrum (cf. the red line in Supplementary Figure 10f), the splitting of 2D peaks is absent, as shown in Supplementary Figure 10c (see cross sections). We found that these results do not depend on the choice of the rates of pure dephasing noise and relaxation and also the vibronic coupling strengths quantified by $S_{\mathrm{X}}, S_{\mathrm{P}}, S_{\mathrm{P*}}$. In addition, the incoherent model does not predict low-frequency components ($< 1000\,\mathrm{cm}^{-1}$) in transients, as shown in Supplementary Figure 10d and e, contrary to the results of the coherent model (cf. Fig. 3 in the manuscript and Supplementary Figure 9). These results support our conclusions that the splitting of 2D peaks and multi-frequency transients observed in experiments are the evidence of coherent couplings in the present system. We note that the model parameters estimated in this work have a significant uncertainty as there are many different sets of model parameters that reproduce the absorption spectrum quite well and also induce the splitting of homogeneously broadened 2D peaks in the absence of inhomogeneous

broadening. For all cases, we found that coherent electronic and vibronic couplings are required with different values of S_X and S_P to obtain the above mentioned features within our phenomenological model: a sufficiently large HR factor S_X of the exciton state is required to reproduce strong vibrational progressions in the absorption spectrum, while a moderate difference in S_X and S_P and other model parameters in an appropriate parameter regime are required to reproduce the splitting of 2D peaks observed in experiments. We note that the splitting of 2D peaks is robust against the variation of model parameters in simulations, due to the strong electronic and vibronic couplings of the present system (cf. Supplementary Table 2).

Incoherent polaron pair formation in presence of two vibrational modes. Up to now we restricted our discussion to models incorporating only one vibrational. The Raman spectrum of P3HT shows a weak vibrational mode around 730 cm^{-1}, corresponding to the C-S-C deformation of the thiophene ring[11, 12]. We have detected this mode also in the Raman spectrum of polymer samples with the same regioregularity, molecular weight and same processing conditions as those investigated in the present work[7]. The mode is also seen in the transient absorption dynamics (insets in Supplementary Figure 3). The presence of this additional mode might not be evident in the absorption spectrum but it could give rise to diagonal peaks in the 2DES maps around 2.15 eV. In order to check if the observed experimental peak splittings in the 2DES maps are the mere superposition of the response of two vibrational modes coupled to the electronic transitions, we performed simulations of the 2DES maps and dynamics in which two vibrational modes (one at 1450 cm^{-1} and another one at 730 cm^{-1}) are coupled to the electronic states.

We assume in this scenario that the electronic coupling between exciton and polaron pair is in the weak coupling limit. In this model, we first deduce a maximum Huang-Rhys factor of 0.05 for the 730 cm^{-1} mode by analyzing Fourier transforms of pump-probe dynamics such as

those shown in Supplementary Figure 3. We then calculate 2DES maps and observe that these show the characteristic peaks of the incoherent exciton model (Supplementary Figure 11a). A characteristic peak splitting, such as the one shown in Fig. 1 of the manuscript, is not observed (Supplementary Figure 11a-b). In order for the 730 cm^{-1} mode to induce the peak splittings observed in our experiment, the Huang-Rhys factor should exceed 1 (Supplementary Figure 11c). In this case, however, the dynamics of all peaks would show large and long-lived beatings along the waiting time (Supplementary Figure 11d). These strong beatings are clearly not detected in our measured 2DES experiment (Fig. 2). Hence, we conclude that the Huang-Rhys factor of the 730 cm^{-1} vibrational mode is too weak to account for the experimentally observed peak splittings.

7. Quantification of mixing between exciton, polaron pair and C=C stretch mode

Displaced vibrational basis. To clarify the relation between the observed splitting of 2D peaks and the coherent mixing between exciton and polaron pair states, we analyze the nature of the vibronic states associated with the homogeneously broadened peaks. To this end, we consider the Hamiltonian in the displaced vibrational basis defined by $\tilde{\mathrm{H}} = \mathrm{UHU}^{\dagger}$ with the unitary operator given by $\mathrm{U}^{\dagger} = |\mathrm{G}\rangle\langle\mathrm{G}| \otimes \mathrm{D}^{\dagger}(0) + \sum_{\mathrm{k=X,P,P*}} |\mathrm{k}\rangle\langle\mathrm{k}| \otimes \mathrm{D}^{\dagger}(\sqrt{S_{\mathrm{k}}})$. In this basis, the vibrational mode is specified in terms of displaced Fock states. Due to the properties of the unitary displacement operator $\mathrm{D}(x)\mathrm{a}\mathrm{D}^{\dagger}(x) = \mathrm{a} - x$ and $\mathrm{D}(x)\mathrm{a}^{\dagger}\mathrm{D}^{\dagger}(x) = \mathrm{a}^{\dagger} - x$, the Hamiltonian $\tilde{\mathrm{H}} = \mathrm{UHU}^{\dagger}$ in the displaced basis is reduced to

$$\begin{aligned}\tilde{\mathrm{H}} = {} & |\mathrm{G}\rangle\langle\mathrm{G}|(\hbar\omega \mathrm{a}^{\dagger}\mathrm{a}) + |\mathrm{X}\rangle\langle\mathrm{X}|(\hbar\Omega_{\mathrm{X}} + \hbar\omega \mathrm{a}^{\dagger}\mathrm{a}) + |\mathrm{P}\rangle\langle\mathrm{P}|(\hbar\Omega_{\mathrm{P}} + \hbar\omega \mathrm{a}^{\dagger}\mathrm{a}) \\ & + |\mathrm{X}\rangle\langle\mathrm{P}|\mathrm{D}(\sqrt{S_{\mathrm{X}}})(\hbar J + \hbar\omega\sqrt{S_{\mathrm{XP}}}(\mathrm{a}^{\dagger} + \mathrm{a}))\mathrm{D}^{\dagger}(\sqrt{S_{\mathrm{P}}}) \\ & + |\mathrm{P}\rangle\langle\mathrm{X}|\mathrm{D}(\sqrt{S_{\mathrm{P}}})(\hbar J + \hbar\omega\sqrt{S_{\mathrm{XP}}}(\mathrm{a}^{\dagger} + \mathrm{a}))\mathrm{D}^{\dagger}(\sqrt{S_{\mathrm{X}}}) + |\mathrm{P}^*\rangle\langle\mathrm{P}^*|(\hbar\Omega_{\mathrm{P}^*} + \hbar\omega \mathrm{a}^{\dagger}\mathrm{a}).\end{aligned} \tag{17}$$

Here the vibrational energy term $\hbar\omega a^{\dagger}a$ describes the quantized vibrational levels of the C=C stretch mode in each electronic state manifold. The time evolution of the vibronic state $\tilde{\rho}(t) = U\rho(t)U^{\dagger}$ in the displaced basis is governed by a master equation that is also of the Lindblad form. We can therefore write

$$\frac{d\tilde{\rho}(t)}{dt} = -\frac{i}{\hbar}[\tilde{H}, \tilde{\rho}(t)] + L_{dep}[\tilde{\rho}(t)] + \tilde{L}_{rxn}[\tilde{\rho}(t)] + \tilde{L}_{vib}[\tilde{\rho}(t)], \quad (18)$$

where the electronic and vibrational relaxation terms are given by

$$\tilde{L}_{rxn}[\tilde{\rho}(t)] = \gamma_{rxn}\left(|P\rangle\langle X| \otimes D(\sqrt{S_P} - \sqrt{S_X})\tilde{\rho}(t)|X\rangle\langle P| \otimes D^{\dagger}(\sqrt{S_P} - \sqrt{S_X}) - \frac{1}{2}\{|X\rangle\langle X|, \tilde{\rho}(t)\}\right), \quad (19)$$

$$\tilde{L}_{vib}[\tilde{\rho}(t)] = \gamma_{vib}\left(2a\tilde{\rho}(t)a^{\dagger} - \{a^{\dagger}a, \tilde{\rho}(t)\}\right). \quad (20)$$

In the displaced basis, the transition dipole operators describing the optical transition between ground state and exciton can be expressed as follows

$$\tilde{\mu}_X^{+} = U\mu_X^{+}U^{\dagger} = \hat{\mathbf{e}}\cdot\vec{\boldsymbol{\mu}}_X |X\rangle\langle G| \otimes D(\sqrt{S_X}), \tilde{\mu}_X^{-} = U\mu_X^{-}U^{\dagger} = \hat{\mathbf{e}}\cdot\vec{\boldsymbol{\mu}}_X |G\rangle\langle X| \otimes D^{\dagger}(\sqrt{S_X}), \quad (21)$$

where the displacement operator $D(\sqrt{S_X})$ describes the Franck-Condon (FC) factors between vibrational levels in ground state and those in exciton state manifold, denoted by $|G,n\rangle$ and $|X,n\rangle$, respectively: here $n = 0, 1, 2, \cdots$, and the vibrational levels in electronic state $|k\rangle$ are defined by $\left(|k\rangle\langle k| \otimes \hbar\omega a^{\dagger}a\right)|k,n\rangle = \hbar\omega n|k,n\rangle$. For instance, the transition dipole strength between $|G,0\rangle$ and $|X,n\rangle$, the so-called $0\text{-}n$ transition, is described by a coefficient $\langle n|D(\sqrt{S_X})|0\rangle$.

Bright vibronic eigenstates of the Hamiltonian. Due to the high frequency of the C=C stretch mode, which is approximately seven times higher than the thermal energy at room temperature, the initial state of the vibronic system is well approximated by $\left|G,0\right\rangle$. In the displaced basis, the vibronic eigenstates of the Hamiltonian are formally expressed as $\left|XP_k\right\rangle = \sum_{n=0}^{\infty}(\psi_{X,n}^{(k)}\left|X,n\right\rangle + \psi_{P,n}^{(k)}\left|P,n\right\rangle)$ defined by $\tilde{H}\left|XP_k\right\rangle = \hbar\Phi_k\left|XP_k\right\rangle$ with the associated energy level of Φ_k. The energy-level difference between $\left|XP_k\right\rangle$ and $\left|G,0\right\rangle$, given by Φ_k, determines the location of the absorptive or 2D peak originating from the optical transition from $\left|G,0\right\rangle$ to $\left|XP_k\right\rangle$.

For the model parameters used in Fig. 3 of the manuscript (cf. Supplementary Table 2), Supplementary Table 5 shows the energy levels of the vibronic eigenstates and their amplitudes $\psi_{X,n}^{(k)}$ and $\psi_{P,n}^{(k)}$ in the displaced basis. It is notable that all the vibronic eigenstates have amplitudes in both exciton and polaron pair bases, i.e. $\left|X,n\right\rangle$ and $\left|P,n\right\rangle$, implying that these vibronic states are coherent superpositions of exciton and polaron pair states. From the fact that the optical transition from ground state to polaron pair is forbidden in our model, the transition from ground state to a vibronic state is optically allowed if and only if the vibronic state has amplitudes in the exciton basis. This coherent mixing or coherent superposition of exciton and polaron pair states leads to dipole borrowing effects, such that all the vibronic states have moderate transition dipole strengths. As shown in Supplementary Table 5, the energy levels Φ_k of the vibronic states are well matched to the location of homogeneously broadened peaks in absorption spectrum (cf. Fig. 3f) and homogeneously broadened 2D peaks (cf. Fig. 3b, c), which are close to the location of experimentally observed splitting of 2D spectra.

In order to quantify the degree of coherent mixing of exciton and polaron pair states in each vibronic eigenstate $|\mathrm{XP_k}\rangle$, we introduce an operator $\sigma_z = |\mathrm{P}\rangle\langle\mathrm{P}| - |\mathrm{X}\rangle\langle\mathrm{X}|$. The expectation value of σ_z shows the relative amplitudes in exciton and polaron pair states, which ranges from -1 (for a pure exciton state) to 1 (for a pure polaron pair state). An expectation value $\langle\sigma_z\rangle$ close to 0 indicates strong mixing of exciton and polaron pair. Here we quantify the mixing of exciton and polaron pair by using the variance of σ_z, defined by $\mathrm{Var}(\sigma_z) = 1 - \langle\sigma_z\rangle^2 = 4\rho_{\mathrm{XX}}^{(k)}(1 - \rho_{\mathrm{XX}}^{(k)})$ where $\rho_e^{(k)} = \mathrm{Tr}_v[|\mathrm{XP_k}\rangle\langle\mathrm{XP_k}|] = \sum_{\mathrm{i,j=X,P}} \rho_{\mathrm{ij}}^{(k)} |\mathrm{i}\rangle\langle\mathrm{j}|$ denotes the reduced electronic state of the vibronic eigenstate $|\mathrm{XP_k}\rangle$. In Supplementary Table 5, the degree of exciton-polaron-pair mixing of the vibronic eigenstates is displayed.

In Supplementary Table 5, the first two dominant amplitudes of each vibronic state in the displaced basis are marked in red, where the first two lowest energy vibronic states are approximately given by $|\mathrm{XP_{k=1,2}}\rangle \approx \psi_{\mathrm{X},0}^{(k)}|\mathrm{X},0\rangle + \psi_{\mathrm{P},1}^{(k)}|\mathrm{P},1\rangle$, while the higher energy vibronic states are approximately given by $|\mathrm{XP_{k=3,4}}\rangle \approx \psi_{\mathrm{X},1}^{(k)}|\mathrm{X},1\rangle + \psi_{\mathrm{P},2}^{(k)}|\mathrm{P},2\rangle$, because the electronic energy of the polaron pair state is lower than the exciton energy (see Supplementary Table 2). These results show that the vibronic states have entanglement (i.e. a form of quantum correlations) between electronic and vibrational degrees of freedom, as shown in Supplementary Table 5 where the entanglement of each vibronic state is quantified in terms of the von Neumann entropy of the reduced density matrix of the electronic degrees of freedom[13, 14], defined by $-\mathrm{Tr}[\rho_e^{(k)}\log_2[\rho_e^{(k)}]] = -\sum_{i=1}^{2}\lambda_i \log_2[\lambda_i]$, where λ_i denote the eigenvalues of the reduced density matrix of the electronic degrees of freedom $\rho_e^{(k)}$ of the vibronic state $|\mathrm{XP_k}\rangle$, given by $\lambda_i = \frac{1}{2}(1 + (-1)^i\sqrt{4\left(\rho_{\mathrm{XX}}^{(k)}\right)^2 + 4\left|\rho_{\mathrm{XP}}^{(k)}\right|^2 - 4\rho_{\mathrm{XX}}^{(k)} + 1})$. This implies

that all the vibronic states cannot be written as product states in the form of $(\psi_{\mathrm{X}}|\mathrm{X}\rangle+\psi_{\mathrm{P}}|\mathrm{P}\rangle)\otimes|\phi\rangle$ with a vibrational state $|\phi\rangle$, indicating that the vibronic states are the results of the coherent mixing among exciton, polaron pair and vibrational degrees of freedom due to the coherent electronic and vibronic couplings.

We note that in the absence of coherent electronic and vibronic couplings, the coherent mixing between exciton and polaron pair states is absent, for which the eigenstates of the Hamiltonian consist of bright exciton states $(\psi_{\mathrm{P,n}}^{(\mathrm{k})}=0)$ and dark polaron pair states $(\psi_{\mathrm{X,n}}^{(\mathrm{k})}=0)$, which do not lead to the splitting of absorptive and 2D peaks in the simulations (cf. Supplementary Figure 10).

The mixing of electronic and vibrational degrees of freedom essentially results from the fact that the electronic and vibronic couplings in the Hamiltonian are diagonalized in different bases. In order to see this in more detail, we consider the electronic Hamiltonian

$$\begin{aligned}\mathrm{H}_{\mathrm{e}}=&\hbar(\Omega_{\mathrm{X}}+\omega S_{\mathrm{X}})|\mathrm{X}\rangle\langle\mathrm{X}|+\hbar(\Omega_{\mathrm{P}}+\omega S_{\mathrm{P}})|\mathrm{P}\rangle\langle\mathrm{P}|\\&+\hbar\mathrm{J}(|\mathrm{X}\rangle\langle\mathrm{P}|+|\mathrm{P}\rangle\langle\mathrm{X}|)+\hbar(\Omega_{\mathrm{P*}}+\omega S_{\mathrm{P*}})|\mathrm{P}*\rangle\langle\mathrm{P}*|.\end{aligned}\tag{22}$$

The eigenstates of the electronic Hamiltonian H_{e} are given by

$$|\Theta_1^{(\mathrm{e})}\rangle=|\mathrm{G}\rangle,\tag{23}$$

$$|\Theta_2^{(\mathrm{e})}\rangle=\frac{2J|\mathrm{X}\rangle+(\delta+(4J^2+\delta^2)^{1/2})|\mathrm{P}\rangle}{\sqrt{2}(4J^2+\delta^2)^{1/4}[(4J^2+\delta^2)^{1/2}+\delta]^{1/2}},\tag{24}$$

$$|\Theta_3^{(\mathrm{e})}\rangle=\frac{(\delta+(4J^2+\delta^2)^{1/2})|\mathrm{X}\rangle-2J|\mathrm{P}\rangle}{\sqrt{2}(4J^2+\delta^2)^{1/4}[(4J^2+\delta^2)^{1/2}+\delta]^{1/2}},\tag{25}$$

$$|\Theta_4^{(\mathrm{e})}\rangle=|\mathrm{P}*\rangle.\tag{26}$$

where $\delta=\Omega_{\mathrm{P}}-\Omega_{\mathrm{X}}+\omega(S_{\mathrm{P}}-S_{\mathrm{X}})$. In this electronic basis, the other terms $\mathrm{H}-\mathrm{H}_{\mathrm{e}}$ of the total Hamiltonian H have off-diagonal components as shown below

$$\sum_{i\neq j}\left|\Theta_i^{(e)}\right\rangle\left\langle\Theta_i^{(e)}\right|\left(\mathrm{H}-\mathrm{H}_e\right)\left|\Theta_j^{(e)}\right\rangle\left\langle\Theta_j^{(e)}\right|$$
$$=\frac{\hbar\omega(a^\dagger+a)}{(4J^2+\delta^2)^{1/2}}\left(\left|\Theta_2^{(e)}\right\rangle\left\langle\Theta_3^{(e)}\right|+\left|\Theta_3^{(e)}\right\rangle\left\langle\Theta_2^{(e)}\right|\right)\left(J(\sqrt{S_X}-\sqrt{S_P})+\delta\sqrt{S_{XP}}\right). \quad (27)$$

This implies that the vibronic coupling terms are not diagonalized in the electronic eigenstates, which makes the vibronic eigenstates of the total Hamiltonian have a mixing between electronic and vibrational degrees of freedom.

These results demonstrate that coherent electronic and vibronic couplings can induce a coherent mixing of exciton, polaron pair and vibrational degrees of freedom, leading to bright vibronic eigenstates of the Hamiltonian. The degree of mixing can be affected by decoherence as well as inhomogeneous broadening, but the presence of 2D peak splitting in both experimental and simulated results demonstrates that the mixing is robust against the noise and disorder in our experimental setup.

8. The role of non-equilibrium vibrational motion in polaron pair formation.

With the model parameters estimated based on experimentally measured absorption and 2D spectra, we now investigate the time evolution of the vibronic system and study the role of non-equilibrium vibrational motion of the C=C stretch mode in the polaron pair dynamics. Here we take the initial state of the vibronic system to be $|X\rangle\otimes|0_G\rangle$ where $|0_G\rangle$ is the vibrational ground state in the electronic ground state manifold. The vertical optical transition from ground state to the exciton state leads to such an initial state.

In Supplementary Figure 12a, we consider the case in which the polaron pair dynamics is governed by coherent couplings in the absence of relaxation (cf. $J\neq 0, S_{XP}\neq 0, \gamma_{rxn}=0$, see the model parameters summarized in Supplementary Table 3). Here the population dynamics

of the polaron pair state (shown in black) shows oscillatory behavior with a period of ~50 fs, which is close to the frequency of the low-frequency oscillations ($< 1000\,\mathrm{cm}^{-1}$) shown in Supplementary Figure 9d, e. The electronic coherence between exciton and polaron pair, which is quantified by $|\langle \mathrm{X}|\rho_{\mathrm{e}}(t)|\mathrm{P}\rangle| + |\langle \mathrm{P}|\rho_{\mathrm{e}}(t)|\mathrm{X}\rangle|$ for the reduced electronic state $\rho_{\mathrm{e}}(t)$, is shown in blue, while the entanglement between electronic and vibrational degrees of freedom, quantified by the logarithmic negativity[15], is displayed in red. We note that due to the pure dephasing noise, the initial vibronic state becomes a statistical mixture of pure states, called a mixed state, for which the logarithmic negativity is the most suitable and easy to compute measure that quantifies the degree of entanglement. While it also applies to the pure state case, there it is customary to employ the von Neumann entropy due to its explicit operational meaning (cf. Supplementary Table 5). These results show the coherent features in the polaron pair rise dynamics. In particular, the non-zero entanglement implies that vibrational motion is quantum-mechanically correlated with electronic motion.

In Supplementary Figure 12b, we assume that the vibrational state of the C=C stretch mode does not change in time. In other words, the vibronic state is taken to be $\rho(t) = \rho_{\mathrm{e}}(t) \otimes |0_{\mathrm{G}}\rangle\langle 0_{\mathrm{G}}|$ for all times t in simulations. Here the initial population dynamics of the polaron pair state is similar to the case of Supplementary Figure 12a up to ~5 fs, but the subsequent dynamics are significantly different. When the non-equilibrium vibrational motion is not allowed to participate in the evolution, the time scale of the polaron pair rise is significantly suppressed. These results originate from the fact that the vibrational motion can modulate the resonance, i.e. the energy-level difference, between exciton and polaron pair states significantly due to the large HR factors of the present system. When the vibrational motion is hindered, the exciton and polaron pair states are non-resonant in energy, leading to slower polaron pair rise. Here the quantum correlations between electronic and vibrational

degrees of freedom are absent (no entanglement), as electronic motion is decoupled from vibrational motion.

In Supplementary Figure 12c and d, we consider the parameters used in Supplementary Figure 12a and b, respectively, but inhomogeneous broadening of the electronic energy difference between exciton and polaron pair is now included by means of considering a Gaussian distribution with a FWHM of 200 meV. It is notable that even in the presence of a large disorder in the electronic energy difference, the overall system dynamics are not changed significantly due to the strong electronic and vibronic couplings.

Supplementary Figure 12e-h shows the case that both pure dephasing noise and relaxation are present (cf. $J \neq 0, S_{\mathrm{XP}} \neq 0, \gamma_{\mathrm{rxn}} \neq 0$, see the model parameters summarized in Supplementary Table 2). Due to the incoherent relaxation process, the time scale of the polaron pair rise is faster than a-d. It is notable that the non-equilibrium vibrational motion enhances the polaron pair rise at initial times (cf. e) compared to the case that vibrational motion is not allowed (cf. f), and these features are maintained even in the presence of the inhomogeneous broadening of the electronic energy difference with a FWHM of 200 meV (cf. g and h).

These results suggest that the non-equilibrium vibrational motion of the C=C stretch mode can modulate the polaron pair rise dynamics significantly by dynamically tuning the energy-level difference between exciton and polaron pair states. Such a dynamic tuning seems to play an important role here as the present system has large HR factors, leading to significant modulation of the degree of resonance in coherent population transfer dynamics (See also Example in Fig. 5 of ref. 16). This is similar to the theoretical results for a blend based on time-dependent density functional theory, where the vibrational motion of the C=C stretch mode is shown to modulate the energy-level difference between exciton and charge-transfer (CT) states, governing the dynamics of the CT state population[17]. The present results, based on the model parameters estimated from experimental data, demonstrate that such vibration-

induced dynamic resonance energy transfer could be possible even in the presence of high decoherence rates and large static disorder of the organic photovoltaics.

9. Acceleration of polaron pair formation due to vibronic coupling

In the absence of vibronic coupling, the dynamics of exciton $|\mathrm{X}\rangle$ and polaron pair $|\mathrm{P}\rangle$ states is equivalent to that of two coupled electronic states with the vibrational mode being a simple spectator. Thus, if we assume that the excitation pulse initially prepares the system in the $|\mathrm{X}\rangle$ state, we will observe population oscillations between $|\mathrm{X}\rangle$ and $|\mathrm{P}\rangle$ with a period $T = \frac{\pi}{\Omega_{\mathrm{R}}}$, where $\hbar\Omega_{\mathrm{R}} = \frac{1}{2}\sqrt{4J^2 + \Delta E^2}$ is the Rabi energy defined by the electronic coupling strength J and the energetic detuning ΔE between $|\mathrm{X}\rangle$ and $|\mathrm{P}\rangle$ states. The dynamics of the polaron pair population can be described as

$$\left|\mathrm{p}(t)\right|^2 \approx \frac{4J^2}{4J^2 + \Delta E^2} \sin^2\left[\sqrt{4J^2 + \Delta E^2}\,\frac{t}{2\hbar}\right]$$

From this equation we can see that the maximum oscillation amplitude is reached when the bare uncoupled states $|\mathrm{X}\rangle$ and $|\mathrm{P}\rangle$ are perfectly resonant, i.e. $\Delta E = 0$. In this case, the population oscillates completely between $|\mathrm{X}\rangle$ and $|\mathrm{P}\rangle$ and the transfer rate is maximal. Conversely, any energetic detuning between the bare states will reduce the amplitude of these oscillations while slightly increasing the oscillation frequency.

In the polymer samples, rapid electronic dephasing will quickly dampen these coherent population oscillations. To estimate the dynamics of the polaron pair formation in the absence of vibronic coupling ($\Delta_{\mathrm{XP}} = 0$), we have calculated the population dynamics from the time evolution of the density operator and then estimated the rise time of these dynamics for the

polaron pair state by fitting a simple exponential rise function to the data. We assume an electronic dephasing time of 50 fs for both $|X\rangle$ and $|P\rangle$ states.

We observe that, in presence of dissipation, the populations oscillations are additionally exponentially damped with a rate defined by the electronic dephasing time γ.

As can be seen in Supplementary Figure 13, this population rise time varies from less than 10 fs when the states are resonant (zero detuning) to more than 200 fs for detuning of a few hundred meV. Hence, in this model, the energetic detuning between $|X\rangle$ and $|P\rangle$ has a large effect on the polaron pair formation.

In the polymer samples, the position of exciton and polaron pair states, and thus their relative energetic detuning, is primarily determined by the respective binding energy of the electron-hole pair. DFT studies have reported typical values of detuning of a few hundred meV for conjugated polymers[18, 19]. Additionally, inherent disorder in the samples induces local fluctuations of the state energy which further increases the energetic detuning between $|X\rangle$ and $|P\rangle$ states. Thus, in the absence of vibronic coupling, one might expect that polaron pair formation occurs on much longer time scales of several hundred fs. Another important point to notice is that, in the absence of vibronic coupling, the transfer integral between $|X, \nu_X\rangle$ and $|P, \nu_P\rangle$ states with different vibrational quantum number ν is always zero and is instead unity only for $\Delta\nu_{XP} = \nu_X - \nu_P = 0$. This essentially means that vibrations do not play a role in the dynamics and act as spectator modes.

This is fundamentally different in the presence of vibronic coupling, i.e., in the presence of a finite displacement Δ_{XP} between $|X\rangle$ and $|P\rangle$ states. Hereby, the transfer integrals become nonzero also for states with $\Delta\nu_{XP} \neq 0$ and thus population transfer will be allowed also

between states with different vibrational quanta. The larger Δ_{XP}, the larger is the overall transfer integral between the states and the faster the transfer rate. Physically, this is readily understood. Due to the ladder of vibrational states in the $|X\rangle$ and $|P\rangle$ states, the energetic displacement between a certain donor mode $|X, \nu_X\rangle$ and the nearest acceptor mode $|P, \nu_P\rangle$ can never be more than one half of a vibrational quantum. Hence, the energetic detuning between donor and acceptor has only a very moderate effect on the transfer dynamics. One may also say that, in the coupled (eigenstate) basis, mixing of electronic $|X\rangle$ and $|P\rangle$ states with the vibrational mode, opens up new paths for coherent population transfer through delocalized vibronic states. Thus, in the presence of strong vibronic coupling, rather fast population transfer is possible even for large energetic detuning between the electronic states. In Supplementary Figure 13, we show the polaron pair rise time simulated for various displacements Δ_{XP}. This suggests that strong vibronic coupling indeed speeds up polaron pair formation dynamics even in presence of relatively large disorder. Supplementary Figure 13 shows quite clearly that the vibronic coupling makes the population transfer dynamics insensitive to the precise value of the detuning and hence to the precise local realization of the disorder potential in the polymer sample. Our results suggest that this effect not only occurs in the samples that we have studied in this manuscript but is a general feature of vibronically enhanced charge transfer phenomena. As such, we believe that the efficient initial charge separation observed on ultrafast time scales is relevant not only to this particular conjugated polymer, but is a general result for systems with large electron-phonon couplings.

Supplementary References

1. Österbacka R, An CP, Jiang XM, Vardeny ZV. Two-Dimensional Electronic Excitations in Self-Assembled Conjugated Polymer Nanocrystals. *Science* **287**, 839-842 (2000).

2. Guo J, Ohkita H, Benten H, Ito S. Near-IR Femtosecond Transient Absorption Spectroscopy of Ultrafast Polaron and Triplet Exciton Formation in Polythiophene Films with Different Regioregularities. *J Am Chem Soc* **131**, 16869-16880 (2009).

3. Howard IA, Mauer R, Meister M, Laquai F. Effect of Morphology on Ultrafast Free Carrier Generation in Polythiophene:Fullerene Organic Solar Cells. *J Am Chem Soc* **132**, 14866-14876 (2010).

4. Song Y, Hellmann C, Stingelin N, Scholes GD. The separation of vibrational coherence from ground- and excited-electronic states in P3HT film. *J Chem Phys* **142**, 212410 (2015).

5. Falke SM*, et al.* Coherent ultrafast charge transfer in an organic photovoltaic blend. *Science* **344**, 1001-1005 (2014).

6. Clark J, Silva C, Friend RH, Spano FC. Role of Intermolecular Coupling in the Photophysics of Disordered Organic Semiconductors: Aggregate Emission in Regioregular Polythiophene. *Phys Rev Lett* **98**, 206406 (2007).

7. Falke S, Eravuchira P, Materny A, Lienau C. Raman spectroscopic identification of fullerene inclusions in polymer/fullerene blends. *J Raman Spectrosc* **42**, 1897-1900 (2011).

8. Lim J, Mark T, Ki Hyuk Y, Joong-Sung L, Jinhyoung L. Phonon-induced dynamic resonance energy transfer. *New J Phys* **16**, 053018 (2014).

9. Renger T, Klinger A, Steinecker F, Schmidt am Busch M, Numata J, Müh F. Normal Mode Analysis of the Spectral Density of the Fenna–Matthews–Olson Light-Harvesting Protein: How the Protein Dissipates the Excess Energy of Excitons. *J Phys Chem B* **116**, 14565-14580 (2012).

10. Mukamel S. Principles of nonlinear optical spectroscopy. *Oxford University Press* (1995).

11. Baibarac M, Lapkowski M, Pron A, Lefrant S, Baltog I. SERS spectra of poly(3-hexylthiophene) in oxidized and unoxidized states. *J Raman Spectrosc* **29**, 825-832 (1998).

12. Saini V*, et al.* Electrical, Optical, and Morphological Properties of P3HT-MWNT Nanocomposites Prepared by in Situ Polymerization. *J Phys Chem C* **113**, 8023-8029 (2009).

13. Bennett CH, Bernstein HJ, Popescu S, Schumacher B. Concentrating partial entanglement by local operations. *Phys Rev A* **53**, 2046-2052 (1996).

14. Plenio MB, Virmani S. An introduction to entanglement measures. *Quant Inf Comput* **7**, 1-51 (2007).

15. Plenio MB. Logarithmic Negativity: A Full Entanglement Monotone That is not Convex. *Phys Rev Lett* **95**, 090503 (2005).

16. Caruso F, Chin AW, Datta A, Huelga SF, Plenio MB. Highly efficient energy excitation transfer in light-harvesting complexes: The fundamental role of noise-assisted transport. *J Chem Phys* **131**, 105106 (2009).

17. Rozzi CA*, et al.* Quantum coherence controls the charge separation in a prototypical artificial light-harvesting system. *Nat Commun* **4**, 1602 (2013).

18. Borges I*, et al.* Ab Initio Modeling of Excitonic and Charge-Transfer States in Organic Semiconductors: The PTB1/PCBM Low Band Gap System. *J Am Chem Soc* **135**, 18252-18255 (2013).

19. Huix-Rotllant M, Tamura H, Burghardt I. Concurrent Effects of Delocalization and Internal Conversion Tune Charge Separation at Regioregular Polythiophene–Fullerene Heterojunctions. *J Phys Chem Lett* **6**, 1702-1708 (2015).
